# Graphene/MoS_2−x_O_x_/graphene photomemristor with tunable non-volatile responsivities for neuromorphic vision processing

**DOI:** 10.1038/s41377-023-01079-5

**Published:** 2023-02-07

**Authors:** Xiao Fu, Tangxin Li, Bin Cai, Jinshui Miao, Gennady N. Panin, Xinyu Ma, Jinjin Wang, Xiaoyong Jiang, Qing Li, Yi Dong, Chunhui Hao, Juyi Sun, Hangyu Xu, Qixiao Zhao, Mengjia Xia, Bo Song, Fansheng Chen, Xiaoshuang Chen, Wei Lu, Weida Hu

**Affiliations:** 1 School of Physics and Optoelectronic Engineering, Hangzhou Institute for Advanced Study, University of Chinese Academy of Sciences, Hangzhou, 310024 China; 2 State Key Laboratory of Infrared Physics, Shanghai Institute of Technical Physics, Chinese Academy of Sciences, Shanghai, 200083 China; 3 University of Chinese Academy of Sciences, 100049 Beijing, China; 4 Institute of Intelligent Machines, HFIPS, Chinese Academy of Sciences, Hefei, 230031 China; 5 Jianghuai Frontier Technology Coordination and Innovation Center, Hefei, 230088 China; 6 Institute of Microelectronics Technology and High-Purity Materials, Russian Academy of Sciences, Chernogolovka, Moscow district, 142432 Russia

**Keywords:** Optoelectronic devices and components, Photonic devices

## Abstract

Conventional artificial intelligence (AI) machine vision technology, based on the von Neumann architecture, uses separate sensing, computing, and storage units to process huge amounts of vision data generated in sensory terminals. The frequent movement of redundant data between sensors, processors and memory, however, results in high-power consumption and latency. A more efficient approach is to offload some of the memory and computational tasks to sensor elements that can perceive and process the optical signal simultaneously. Here, we proposed a non-volatile photomemristor, in which the reconfigurable responsivity can be modulated by the charge and/or photon flux through it and further stored in the device. The non-volatile photomemristor has a simple two-terminal architecture, in which photoexcited carriers and oxygen-related ions are coupled, leading to a displaced and pinched hysteresis in the current-voltage characteristics. For the first time, non-volatile photomemristors implement computationally complete logic with photoresponse-stateful operations, for which the same photomemristor serves as both a logic gate and memory, using photoresponse as a physical state variable instead of light, voltage and memresistance. The polarity reversal of photomemristors shows great potential for in-memory sensing and computing with feature extraction and image recognition for neuromorphic vision.

## Abstract (web summary)

Two-terminal artificial optoelectronic devices in which photoexcited carriers and ions are coupled constitute a built-in artificial neural network that can sense, memory and process simultaneously.

## Graphical abstract

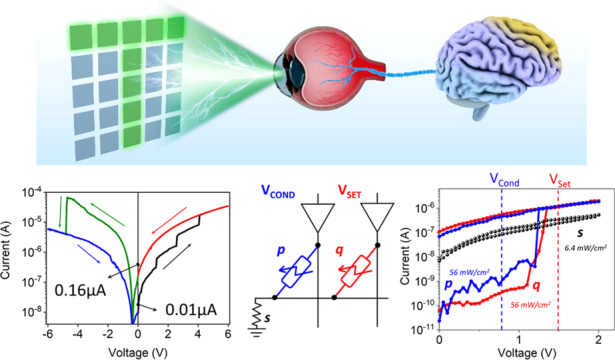

## Introduction

The human vision system has a powerful capability in visual perception only consuming less than twenty watts of power. Such features are mainly attributed to the simultaneous sensing and early processing of visual information in the retina and parallel processing in the visual cortex^1–8^. For example, to efficiently discard the redundant visual data and accelerate subsequent processing tasks in the visual cortex, the human retina can extract critical features of visual data with plastic positive and negative photoresponse^9^. Inspired by the human vision system, AI machine vision technology has been developed to achieve the capability of perception. Usually, in traditional vision systems, the optical information is captured by a frame-based digital camera, and then the digital signal is processed afterward using machine-learning algorithms. In this scenario, a large amount of data (mostly redundant) has to be transferred from a standalone sensing elements to the processing units, which leads to a large latency and power consumption. To address this problem, much effort has been devoted to developing an in-sensor computing technology by emulating certain functions of the human retina, for example, metal-semiconductor-metal variable-sensitivity photodetectors (VSPDs), reconfigurable 2D semiconductor photodiodes, gate-tunable van der Waals heterostructures, etc. The above sensors constitute a built-in artificial neural network that can sense and process images simultaneously^10–17^. However, challenges still exist. The VSPDs based on the metal-semiconductor-metal structure had a bias-dependent dark current, and the reconfigurable 2D material-based neural network image sensors are volatile and need continuing gate voltage to update weights^9,18–21^. To create a non-volatile photodetector with tunable photoresponses requires complex device designs or manufacturing processes, for example, using a floating gate or a ferroelectric gate dielectric. Therefore, in order to efficiently process such a large amount of data and reduce power consumption, it is necessary to develop a non-volatile photodetector device with a simple architecture for high-density integration.

The coupled electron-ion memristive system allows multiple resistive states being adjusted by memorizing the history of previous electrical inputs, thus simulating biological synapses^22–25^. The conductivity of memristors changes with an external bias voltage, while non-volatile resistive states are preserved^22,23,26–29^. Furthermore, the crossbar array based on the memristors can perform the matrix-vector product operation efficiently through Ohm’s law and Kirchhoff’s law with energy-efficient in-memory computing^30,31^. Inspired by memristive devices and the requirement for massively parallel computing in image processing, we have developed simple, two-terminal, non-volatile photomemristors with tunable photoresponsivity, in which the responsivity can be modulated by charge and/or photon flux through it. This new concept provides possibilities to achieve all-in-one sensing-memory-computing device with simple architecture for the implementation of in-sensor computing network.

In this paper, simple two-terminal photomemristors based on 2D Graphene/MoS_2-x_O_x_/Graphene (G/M/G) structures are experimentally demonstrated. Recorded high endurance (more than a hundred cycles) and reliable retention (more than a thousand seconds) indicate that our device can be used for multistate non-volatile photodetection. In addition, we demonstrate the photoresponse-stateful computationally complete logic with the photomemristors set, in which the same photomemristor serves simultaneously as logic gates and memory unit. Instead of physical state variables of light, voltage and conductance^32–34^, the non-volatile photoresponse is first served as the variable. This kind of photoresponse-stateful in-memory computing strategy can expand the functional diversity of edge-side neural networks such as binarized neural networks^33^. Furthermore, the proposed photomemristor arrays provide image pre-processing and recognition with multistate photoresponse, suggesting that a new type of photomemristor opens the possibility for the implementation of an in-sensory network in the future. This new type of two-terminal photomemristor not only provides versatile sensing-memory-computing approaches for neuromorphic vision hardware but also enables high-density integration.

## Results

Graphene/MoS_2-x_O_x_/Graphene (G/M/G) photomemristor structures were fabricated using MoS_2_ nanocrystals (NCs) and CVD-grown graphene as electrodes. MoS_2_ NCs were prepared by the liquid phase exfoliation (LPE) method. The absorption spectrum of the NCs showed 2H-MoS_2_ exciton peaks in the UV-visible region (A, B, C, D). Typical in-plane and out-of-plane vibrational modes of Raman scattering indicated the high quality of the resulting 2H-MoS_2_ phase^26^. Details of structure preparation and material analysis are described in Methods and Supplementary Information (Figs. S1–S6, Supplementary Information).

**Fig. 1:**
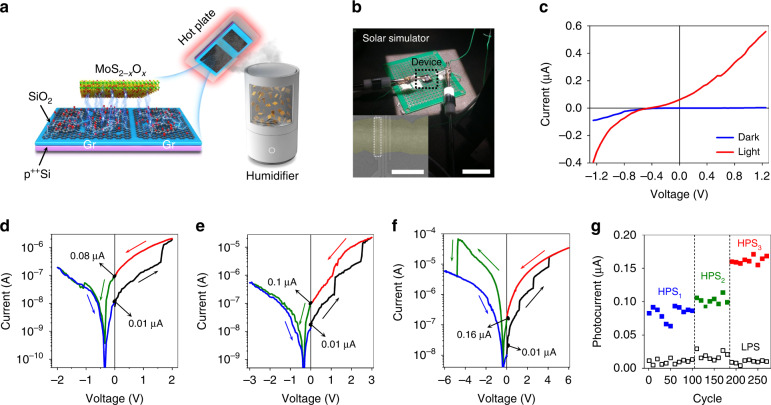
The basic device with displaced and pinched hysteresis current-voltage characteristics. **a** Schematic representation of the MoS_2-x_O_x_ structure with CVD-grown graphene electrodes of different contact areas and the experimental process of LPE film deposition using the ultrasonic deposition setup. Spheres: carbon—black, oxygen—red, molybdenum—green, sulfur—yellow. **b** Optical image of the G/M/G device with solar light illumination. Scale bar, 2 cm. The inset shows an SEM image of another device, scale bar, 250 μm. the white rectangle indicates the area (256.2 × 62.5 μm^2^) of a G/M/G device. **c** Current-voltage characteristics of G/M/G devices at HPS in the dark (blue line) and in the light (red line). **d**–**f** Multilevel photoresponse switching characteristics at a discrete input bias voltage. **g** Endurance of photoresponse states (HPS_1_, HPS_2_, HPS_3_, LPS) over hundreds of switching cycles (reading at 0 V bias)

Figure 1a shows a schematic representation of the MoS_2-x_O_x_ structure with CVD-grown graphene electrodes of different contact areas and an experimental LPE film deposition process using an ultrasonic deposition setup. MoS_2_ NCs were oxidized during deposition at ambient conditions, forming a p-type MoS_2-x_O_x_ thin film in contact with lateral graphene electrodes with asymmetric geometry (Area ratio (SC_2_: SC_1_) ≈ 3.5, thickness of MoS_2-x_O_x_ ≈ 200 nm, Fig. S6, Supplementary Information)^35–37^. It should be noted that CVD-grown graphene contained grain boundaries and bi-graphene islands with an average density of ~3 × 10^6 ^cm^–2,^^38^, which contributed to the oxidation of graphene electrodes during the deposition of MoS_2_ and the formation of MoS_2-x_O_x_ (Figs. S3–S6, Supplementary Information)^38–40^.

The operation of the device was studied by controlling the bias voltage at G/M/G. When a two-terminal asymmetric G/M/G device is illuminated with a solar simulator (power density 56 mW cm^–2^), a photovoltaic effect is observed with a photocurrent (I_L_) to dark current (I_D_) ratio of ~10^3^ at a positive bias (Fig. 1c). Figure 1d shows the photocurrent abruptly switching from 0.29 to 1.23 μA at a SET voltage of 1.60 V during a voltage sweep from 0 to 2 V (sweep rate: 0.05 V s^–1^). When the bias voltage sweeps from 2 V to –2V, the device switches from a high photoresponse state (HPS) to a low photoresponse state (LPS) at a RESET voltage of –1.05 V, demonstrating non-volatile photoresponse memory. Interestingly, the device generates photocurrent without any bias voltage, which is due to the asymmetrical G/M/G contacts, which allows us to read different values of LPS (0.01 μA) and HPS_1_ (0.08 μA) at 0 V bias. A further increase in the sweep voltage to 2.45 V and 4.05 V (Fig. 1e, f) leads to sequential switching of the HPS at a bias of 0 V to 0.1 μA and 0.16 μA, respectively. The retention times for the LPS and HPS states are greater than 3 × 10^3^ s at room temperature, as shown in Fig. S7 (Supplementary Information). Figure 1g shows the readout process at a bias voltage of 0 V with a photocurrent on/off ratio of about 10 for hundreds of cycles (see also Figs. S8 and S9 Supplementary Information showing photocurrents in various switching states and photoresponse states with bias adjustment).

The reliability and performance can be greatly improved when the size of the devices is reduced and the input voltage is increased, as shown in the inset of Fig. 1b and Figs. S10, S11, Table S1 (Supplementary Information). The on/off voltage decreases with decreasing channel length as the voltage fully decreases to completely deplete the semiconductor between the electrodes (Fig. S11d)^41^. In addition, the tunable short-circuit photocurrent and photoresponse can be increased to 889.8 nA and 98.8 mA/W, respectively, which are much higher than other 2D material based phototransistors^19,42^. To reverse the channel polarity and obtain a gate-tunable short-circuit photocurrent, the channel semiconductor must be thin enough. Thus, it is difficult to use the thick film needed to absorb enough light and get a high signal. In our case, the mechanism of the two-terminal device rearrangement is based on ion migration, which does not limit the thickness. We can increase the thickness of the film to absorb more photons and get a high short-circuit photocurrent.

## Discussion

**Fig. 2:**
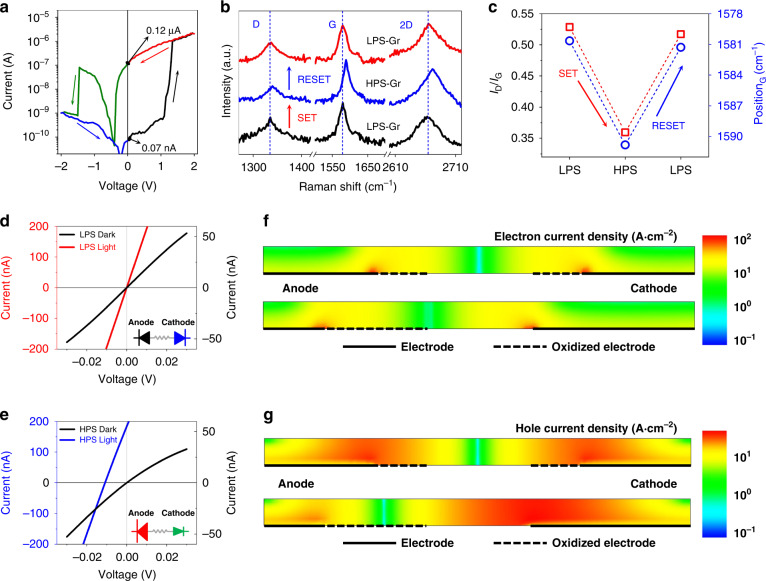
Photoresponse switching mechanism. **a** I-V characteristics of a G/M/G device with binary photoresponse switching when the voltage sweeps from 0 to 2 V, from 2 V to –2 V, and back to 0 V. **b** Raman curves showing the evolution of redox reactions on graphene electrodes when switching between HPS and LPS. **c** Correlation of changes in the ratio of the intensities of Raman scattering and shifts of the G mode of graphene electrodes and states of the non-volatile photoresponse. TCAD-simulated dark/photo current of the G/M/G device for LPS (**d**) and HPS (**e**). The insets of (**d**) and (**e**) demonstrate the qualitative device model for LPS and HPS, respectively. **f** The electron current density distribution of LPS (top) and HPS (bottom) under illumination, simulated with TCAD. **g** TCAD simulated the hole current density distribution of LPS (top) and HPS (bottom) under illumination, simulated with TCAD

In order to investigate the mechanism of non-volatile photoresponsivity switching in two-terminal G/M/G devices, in-situ Raman analysis of structures with thinner MoS_2-x_O_x_ was performed to be able to characterize the underlying graphene electrodes (details in Figs. S12–S15 in Supplementary Information Section C). Figure 2a shows the I-V characteristics of this sample (see also Fig. S12d, Supplementary Information). The photocurrent switches from LPS to HPS at a SET voltage of about 1.2 V, and the HPS switches to LPS at a RESET voltage of about -1.0 V. When the device switched from LPS to HPS, we measured the Raman modes of cathode and anode under MoS_2-x_O_x_. Figure 2b shows the Raman scattering modes of the cathode. The I_D_/I_G_ ratio decreased from 0.51 to 0.33 and the peak positions of the G- and 2D-bands show redshifts of 9 cm^−1^ and 7 cm^−1^, respectively, as shown in Fig. 2b and c. Such a change in the Raman modes indicates the reduction of graphene^43,44^. After the RESET process, the I_D_/I_G_ ratio increased to 0.49. In this case, a blue shift of the G- and 2D-bands was observed, demonstrating the oxidation process^43–45^. At the same time, the corresponding reduction and oxidation of the anode are observed for the Set and Reset processes (see Figs. S14 and S15, Supplementary Information). An increase (decrease) in the short-circuit photocurrent of the device is accompanied by reduction (oxidation) of cathode electrodes, which indicates that photoresponsive switching correlates with reversible redox reactions at the MoS_2-x_O_x_/G interface^46^. Note that such a reversible redox process at the required potential is observed only under illumination. Resistive switching in the dark requires a higher voltage of 12 V (Fig. S16, Supplementary Information).

Reversible partial oxidation (reduction) of graphene (graphene oxide) leads to a reversible decrease (increase) in the mobility of charge carriers by more than an order of magnitude^43^. In addition, Fig. S17 confirms that the conductivity of our CVD graphene decreases when the oxidation degree increases. This can change the collection efficiency of photoexcited carriers at the cathode and anode. To investigate the relationship between photoresponse states and the redox process on graphene electrodes, a series of Sentaurus Technology Computer Aided Design (TCAD) simulations were run as shown in Fig. 2 d–g.

When we read a G/M/G device in a low voltage range, the device acts like two back-to-back Schottky diodes connected in series, as shown in the insets of Fig. 2d and e. As the effective contact size increases (decreases), the resistance of the corresponding diode decreases (increases), and we simplify this change by the diode size^47^. The dark current is symmetric with a symmetrical oxidation degree of cathode and anode as shown in the black curve in Fig. 2d. When the bias voltage is 0, the current density of the anode and cathode have the same values with opposite polarity (top current density plot in Fig. 2f and g) under illumination. Thus, the short-circuit photocurrent is 0, as shown by the red curve in Fig. 2d, which corresponds to the LPS of our G/M/G structure. After applying a positive bias voltage, oxygen vacancies migrate from MoS_2-x_O_x_ to the cathode and from the anode to MoS_2-x_O_x_, leading to the reduction and oxidation of the cathode and anode, respectively. The forward current is less than the reverse current since the resistance of the anode (cathode) has increased (decreased). Under illumination, the TCAD simulated electron/hole current density of the anode and cathode demonstrate different values with opposite polarities, as shown in the bottom current density plots in Fig. 2f and g. The blue curve in Fig. 2e shows a short-circuit photocurrent of about 170 nA, which corresponds to the HPS of our G/M/G structure (Details in Table S2).

To confirm the importance of the observed processes on graphene electrodes in our G/M/G devices for switching their photoresponse, we carried out control experiments on the Au/MoS_2−x_O_x_ (~100 nm)/Au (A/M/A) structure using the same deposition method. Such devices have not shown non-volatile photoresponsivity switching under illumination (Fig. S18, Supplementary Information). These results show that the oxidation and reduction of graphene electrodes play an important role in the non-volatile photoresponsivity switching for our device.

**Fig. 3:**
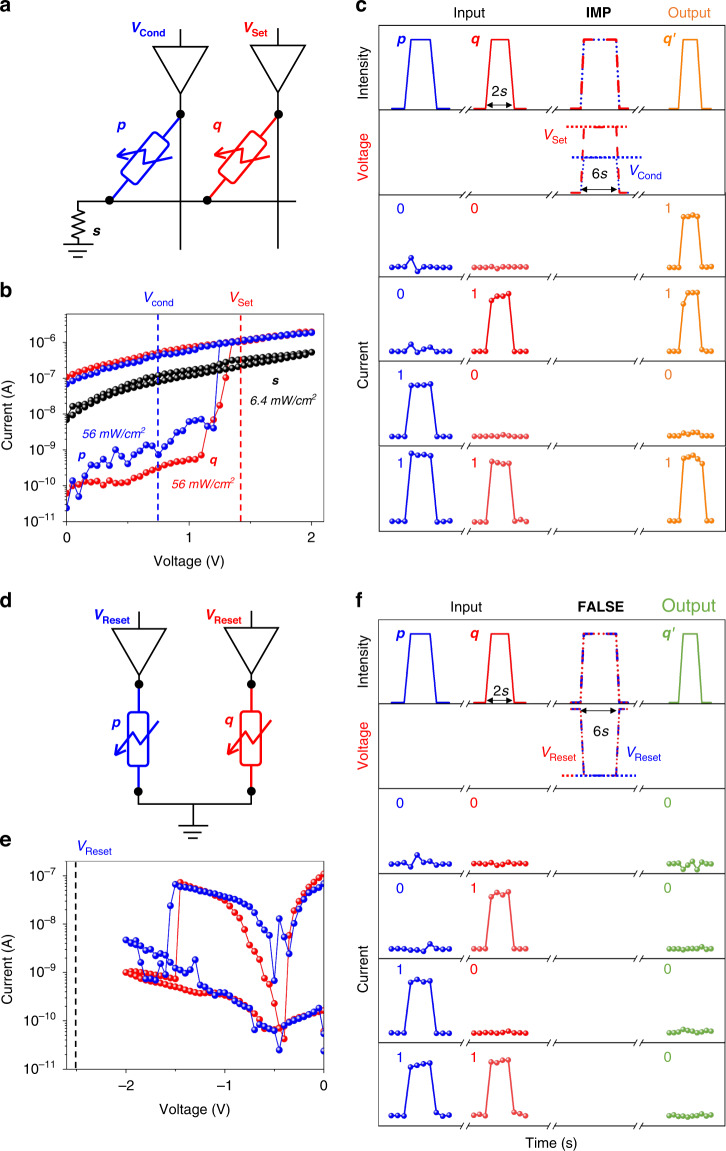
“Photoresponse-stateful” strategy. **a** Illustration of an in-memory IMP operation based on a set of photomemristors triggered by light stimuli. **b** I-V characteristics of devices *s*, *p* and *q* under illumination with different light intensities. **c** Diagram of the optical and electrical pulses applied for the IMP operation. The blue and red curves show the optical and electrical signal of devices *p* and *q* before and during the IMP operation, and the orange curves show the change in photoresponse states after IMP operations, reproducing the IMP truth table. **d** Illustration of an in-memory FALSE operation triggered by light stimuli based on a set of photomemristors. **e** I-V characteristics of devices *p* and *q* under illumination. **f** Diagram of the optical and electrical pulses applied for the FALSE operation. The blue and red curves show the optical and electrical signal of devices *p* and *q* before and during the FALSE operation. The black curves show the change in photoresponse states after FALSE operations, reproducing the FALSE truth table

A computationally complete logical basis, using the fundamental elements of modern digital electronics and optoelectronics, can be formed by material implication (IMP) and FALSE operations (Table S3, Supplementary Information)^32,48^. To implement the IMP operation, a set of three photomemristors *p*, *q* and *s* was developed with the corresponding connection scheme, as shown in Fig. 3a. Figure 3b shows the hysteresis curves of photomemristors for IMP operation. Device *s* is set to HPS and is illuminated during measurements with a light intensity of 6.4 mW cm^-2^, resulting in no photoresponsive switching (Fig. 3b, black curve). The photoconductance of the photomemristor *s* is an order of magnitude lower than that of the photomemristor in an HPS and an order of magnitude higher than that of the photomemristor in an LPS. In this case, devices *p* and *q* are illuminated at a light intensity of 56 mW cm^-2^, which leads to a clear switching of their photoresponse (Fig. 3b).

Figure 3c demonstrates the photoresponse-stateful IMP operation. A positive voltage of 1.5 V (V_SET_) applied for 6 s can switch devices from LPS (defined as 0) to HPS (define as 1), while a lower voltage of 0.8 V (V_COND_) cannot change the photoresponse state. The IMP operation can be implemented by simultaneously applying light and electrical stimulation (V_SET_ to *q* and V_COND_ to *p*) to achieve a conditional photoresponse switching, while reading can be performed by applying light stimuli. Under illumination, the unconditional operation of the set process of device *q* (*q* ← 1) can be executed when V_SET_ and V_COND_ are separately applied to *q* and *p*, respectively. When the *p* photomemristor is in LPS (*p* = 0), V_COND_ has limited influence on the voltage across *q*, thus *q* is set while *p* remains unchanged. If the photomemristor *p* is in HPS (*p* = 1), the voltage applied to *q* is below the threshold voltage at which q must remain without changing its photoresponse state.

Figure 3d–f shows the scheme of photomemristors for implementing a FALSE operation (d) and I-V characteristics with a reset process for *p* and *q* (e). A negative voltage of –2.5 V (V_RESET_) applied for 6 s can toggle the photoresponse state from HPS to LPS. However, the device does not show a negative photoresponse even a –2.5 V pulse is applied to the LPS device (Fig. S19, Supplementary Information). Thus, the FALSE operation can be simply implemented by applying V_RESET_ to *p* and *q* devices under illumination. The results and the corresponding truth table are shown in Fig. 3f. Thus, the IMP and FALSE operations which form the computationally complete logic are performed by G/M/G photomemristors, indicating a promising strategy for performing photoresponse-stateful logic operations triggered together by electrical and light stimuli.

**Fig. 4:**
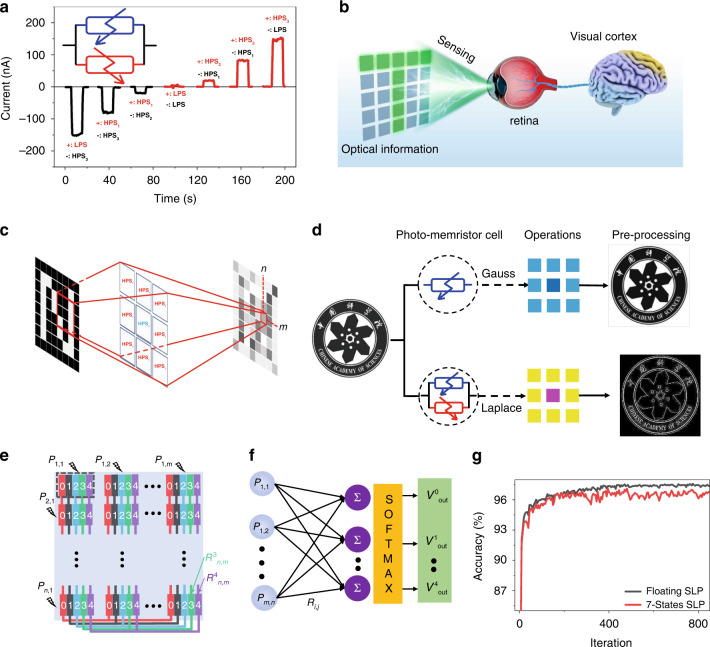
Image pre-processing and classifier based on G/M/G devices. **a** Photocurrent for different photoresponse states, in a different set of photomemristors. The inset shows schematically photomemristors installed with opposite polarity. **b** Schematic representation of the human visual system for sensing, memory, and computing. **c** Photomemristors with integrated sensing-memory-computing architecture. **d** Demonstration of image processing with various operators. These operations are realized by varying the different states and polarities of the photomemristors. **e** Schematic Illustration of the SLP photomemristors array for classifier emulation, all photomemristors with the same class (color) are connected in parallel to obtain the output current for the activation function. **f** SLP neural network architecture schematics. **g** Accuracy of the SLP classifier during training with floating-point weight and discrepant 7-level photoresponse state

The parallel connection of two photomemristors with different polarities allows negative photoresponse states, which provide more freedom for neuromorphic computing functionality. Here we accommodate each device on a printed circuit board and connect them with wire and dial switches, setting a photoresponsive state individually and measuring a set of photomemristors assembled in parallel. Figure 4a shows the 7 distinguishable photoresponse states of our array of photomemristors (Fig. S20, Supplementary Information). Using these 7 states, we emulate two types of neuromorphic vision functions: image pre-processing and classifier. Mimicking the neuromorphic vision preprocessing function of the human retina can speed up subsequent perception tasks and improve the image recognition rate^9^. These G/M/G photomemristors are combined into a 3 × 3 array that allows the simulation of the biological receptive field (RF) of the human retina controlled by different photoresponse states separately. Summing all photocurrents from each photomemristor of the emulated arrays performs the matrix-vector product operation:

1 $${{{\boldsymbol{I}}}}_{{{{\boldsymbol{m}}}},{{{\boldsymbol{n}}}}} = \mathop {\sum}\limits_{i,j}^{3,3} {{{{\boldsymbol{R}}}}_{{{{\boldsymbol{i}}}},{{{\boldsymbol{j}}}}} \times {{{\boldsymbol{P}}}}_{{{{\boldsymbol{i}}}},{{{\boldsymbol{j}}}}}^{{{{\boldsymbol{m}}}},{{{\boldsymbol{n}}}}}}$$

where ***R***_***i,j***_ is the photoresponsivity matrix for various types of kernels and ${{{\boldsymbol{P}}}}_{{{{\boldsymbol{i}}}},{{{\boldsymbol{j}}}}}^{{{{\boldsymbol{m}}}},{{{\boldsymbol{n}}}}}$ is the vector of the optical signal of the input image, as shown in Fig. 4b and c, ***I***_***m,n***_ is the output vector which represents the dynamic current to the input signal. Various types of kernels for image pre-processing can be set by providing various states and polarities of the photomemristor array, allowing the image pre-processing function to be performed. With the working principle, we realized crucial functions that are widely used for image pre-processing with a photomemristor array consisting of a single photomemristor cell, such as the Gaussian blur. When we use photomemristors set as the base cell, as shown in Figs. S20–S23 (Supplementary Information), we can use more complex image pre-processing strategies with distinct kernels, such as the difference of the Gaussian, Prewitt, and Roberts operators. Figure 4d shows the logotype of the Chinese Academy of Sciences by computing a grayscale image using an emulated photomemristor array. Blurred and edge-enhanced images are similar to simulation results (Fig. S21, Supplementary Information). Here, image pre-processing has been achieved without power consumption due to non-volatile photoresponse matrices and signal reading without external biases.

The working principle of the classifier is schematically shown in Fig. 4e. Each cell of the photodetector arrays consists of 5 photodetector sets corresponding to 5 classes (k = 0, 1, 2, 3, 4), summing all photocurrents from the cell with the same class from the emulated arrays performs the matrix-vector product operation:

2 $${{{\boldsymbol{I}}}}_{{{\boldsymbol{k}}}} = \mathop {\sum}\limits_{i,j}^{m,n} {{{{\boldsymbol{R}}}}_{{{{\boldsymbol{i}}}},{{{\boldsymbol{j}}}}}^{{{\boldsymbol{k}}}} \times {{{\boldsymbol{P}}}}_{{{{\boldsymbol{i}}}},{{{\boldsymbol{j}}}}}}$$

where ${{{\boldsymbol{R}}}}_{{{{\boldsymbol{i}}}},{{{\boldsymbol{j}}}}}^{{{\boldsymbol{k}}}}$ is the photoresponsivity matrix for class k, ***P***_***i,j***_ is the vector of the optical signal of the input image, as shown in Fig. 4e and f, the output current ***I***_***k***_ is the input for the activation function. The network consists of a single-layer perceptron (SLP), together with SoftMax functions. This SLP represents a supervised learning algorithm that classifies the input images into 5 classes. The input layer of such SLP captures a 28 × 28-pixel image of 0, 1, 2, 3, 4 from the MNIST dataset, and the fully connected (FC) layer consists of 768 × 10 neurons. SLP is trained offline using 30596 training set images with a batch size 64 and 4000 iterations delivering the final output probability that classifies the input images in the test set (5139 images) into 5 classes with 97.66% accuracy. The weights in FC are discretized to accommodate the 7 photoresponse states. After discretization, the accuracy is about 96.44%, which is 1.22% lower than the pristine SLP. Further emulation of the 10 classes classifier based on our photomemristors demonstrates a 2% reduction in recognition rate compared to the pristine SLP, as shown in Fig. S24 (Supplementary Information). This indicates the potential applications of non-volatile in-sensory computing by using our photomemristors. Thus, here we have demonstrated a non-volatile responsivity matrix for simultaneous perception and processing of visual information using our two-terminal photomemristor without external power consumption.

In summary, we have demonstrated tunable non-volatile photomemristors with a simple two-terminal G/M/G architecture, in which photoexcited carriers and ion migration are coupled leading to a displaced and pinched hysteresis of I-V characteristics. The devices can store and read multiple photoresponse states in a non-volatile mode at zero external voltage. Furthermore, the switching properties can be jointly controlled by the electric-field-driven migration of ions and photo-induced redox reactions at the asymmetric G/M/G contacts. By mimicking the biological functionalities of the human retina and designing specific device structures, the devices can act as a neural network for neuromorphic visual processing and implementation of completely photoresponse-stateful logic operations triggered by electrical and light stimuli together. This new concept of a two-terminal photomemristor not only provides versatile sensing-memory-computing approaches for neuromorphic vision hardware but also enables high-density integration.

## Materials and methods

### Preparation of MoS_2_ nanocrystals

MoS_2_ NCs were obtained using the ultrasonic exfoliation method in the liquid phase. First, 0.6 g of MoS_2_ powder (<2 μm, 99%, Sigma-Aldrich) was dispersed in 60 ml of IPA and 40 ml of deionized water. The dispersion was homogenized in a sealed bottle (110 ml) and exfoliated in an ultrasonic bath (Green Sonic 2000, Woosung Ultrasonic CO. LTD.) for 5 h using ice and circulating water to keep the temperature below 10 °C. Upon completion of the exfoliation step, the dispersion was centrifuged for 30 min at 3000 rpm at 295 K to remove the remaining bulk material and then the top 50% supernatant was carefully collected. After collecting 700 ml of the suspension, it was centrifuged at 8500 rpm for 40 min at 295 K. Then all supernatants were removed, and the sediment was redissolved in 32 ml of methanol and 8 ml of DI water, after which a highly concentrated MoS_2_ NC solution was obtained, to which another 40 ml of DI water was added. As a result, 80 ml (32 ml methanol and 48 ml DI water) of highly concentrated MoS_2_ NC dispersion was obtained in the form of MoS_2_ ink.

### Device fabrication

The electrodes were fabricated from a transferred graphene (Gr) layer grown on Cu-foil by CVD at 1020 °C in a methane/hydrogen flow at 600 mTorr. Photolithography, O_2_ plasma treatment and a lift-off process were used to fabricate the device. The sample with Gr electrodes was fixed on a hot plate with a mask of a given geometry. After preliminary heat treatment of the sample at 210 °C for 20 min, a MUJI humidifier filled with MoS_2_ ink was used to obtain the MoS_2_ film, as shown in Fig S1b. After 70 min of deposition at a temperature of 210 °C, a thin MoS_(2-x)_O_x_ film with a thickness of about 200 nm (Inset in Fig. S6a, Supplementary Information) was formed, in contact with two asymmetric lateral Gr electrodes. Then the sample was kept in a hot plate for another 15 min, followed by an 8-h cleaning process in a vacuum chamber.

### Device characterization

MoS_2_ NCs were analyzed using an FEI Titan G2 60–300 transmission electron microscope operating at a voltage of 200 kV. Optical measurements of MoS_2_ NC were performed using a Varian Cary UV-vis. spectrometer in the 250–1000 nm wavelength range. Raman measurements were carried out using a micro-Raman spectrometer at an excitation wavelength of 532 nm at 295 K. The current-voltage characteristics were investigated using a Keithley 617 semiconductor parameter analyzer. The solar simulator (Newport, AM 1.5) was used to study the photomemristive switching of the device under illumination.

### Details of datasets

We emulate photomemristor arrays by calculating the average photocurrent of each memresponsor at various photoresponse states as shown in Figs. S7 and S20 (Supplementary Information). In Fig. 4, we down-sampled the logotype of the Chinese Academy of Sciences into 386 × 391 Gray Scale Image, the experimental results were calculated by corresponding photomemristor arrays using convolution operation in MATLAB, while the simulation results were calculated by a standard matrix as shown in Figs. S21–S23 (Supplementary Information).

## Supplementary information

Supplementary Information for Graphene/MoS2−xOx/graphene photomemristor with tunable non-volatile responsivities for neuromorphic vision processing
