# Supplementary material for: Graphene/MoS2−xOx/graphene photomemristor with tunable non-volatile responsivities for neuromorphic vision processing
Source: Light Sci Appl. 2023 Feb 7;12:39. doi: 10.1038/s41377-023-01079-5 (PMC9905593; doi:10.1038/s41377-023-01079-5)
Supplement: Supplementary file 1 — Supplementary Information for Graphene/MoS2−xOx/graphene photomemristor with tunable non-volatile responsivities for neuromorphic vision processing [file 41377_2023_1079_MOESM1_ESM.docx]

Supplementary Information for

**Graphene/MoS_2−x_O_x_/graphene photomemristor with tunable non-volatile responsivities for neuromorphic vision processing**

Xiao Fu^a,b,c#^, Tangxin Li^b,c #^, Bin Cai^d,f #^, Jinshui Miao^a,b,c*^, Gennady N. Panin^e^, Xinyu Ma^b,c^, Jinjin Wang^b,c^, Xiaoyong Jiang^b,c^, Qing Li^a,c^, Yi Dong ^b,c^, Chunhui Hao^a,c^, Juyi Sun^a,c^, Hangyu Xu ^b,c^, Qixiao Zhao ^b,c^, Mengjia Xia ^b,c^, Bo Song^d,f*^, Fansheng Chen ^b,c^, Xiaoshuang Chen ^b,c^, Wei Lu ^b,c^, Weida Hu^a,b,c*^

^a^School of Physics and Optoelectronic Engineering, Hangzhou Institute for Advanced Study, University of Chinese Academy of Sciences, Hangzhou 310024, China.

^b^State Key Laboratory of Infrared Physics, Shanghai Institute of Technical Physics, Chinese Academy of Sciences, Shanghai 200083, China.

^c^University of Chinese Academy of Sciences, Beijing 100049, China.

^d^Institute of Intelligent Machines, HFIPS, Chinese Academy of Sciences, Hefei 230031, China.

^e^Institute of Microelectronics Technology and High-Purity Materials, Russian Academy of Sciences, Chernogolovka, Moscow district 142432, Russia.

^f^Jianghuai Frontier Technology Coordination and Innovation Center, Hefei 230088, China

**^*^Corresponding authors**

E-mail: Weida Hu: ([wdhu@mail.sitp.ac.cn](mailto:wdhu@mail.sitp.ac.cn)) or Jinshui Miao: ([jsmiao@mail.sitp.ac.cn](mailto:jsmiao@mail.sitp.ac.cn)) or Bo Song: ([songbo@iim.ac.cn](mailto:songbo@iim.ac.cn)).

^#^These authors contributed equally to the work.

**Table of contents**

**Section A**: Material Preparation and Device Fabrication

**Section B:** Current-Voltage Measurements

**Section C:** Raman characterization

**Section D:** Implementation of stateful logical operations with photomemristors

**Section E:** Emulation of a retinomorphic vision sensor and logical operations with photomemristors


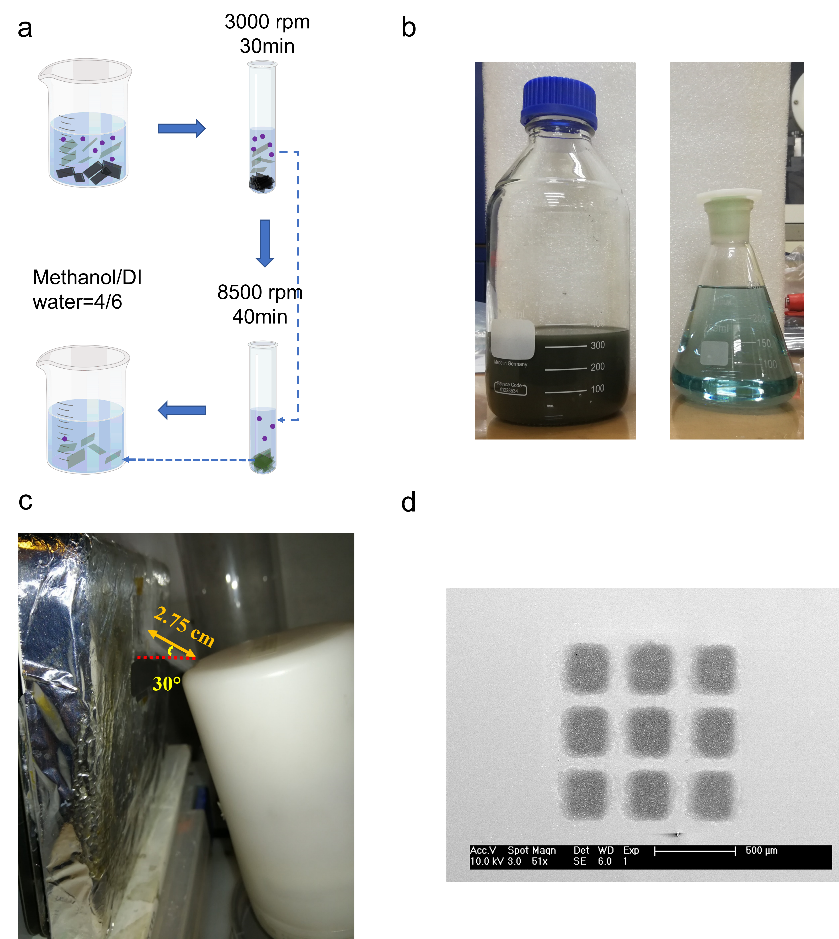
**Section A: Material Preparation and Device Fabrication**

**Figure S1.** (a) Schematic representation of the experimental LPE MoS_2_ process and MoS_2_ ink preparation. (b) Photo of exfoliated NCs (left) and QDs (right). (c) Photo of the ultrasonic deposition process. (d) SEM image of deposited arrays.

**Figure S1a** shows the experimental LPE process. MoS_2-x_O_x_ thin film was deposited at 210°C for 90 min in ambient air using the ultrasonic deposition method as shown in Figure S1c. A thin film of MoS_(2-x)_O_x_ with a thickness of about 200 nm in contact with two lateral graphene electrodes was formed through a mask with a given geometry. For such a device, the channel length is 6 µm, and the contact area of SC_1_ and SC_2_ is 3.5×1.4 mm^2^ and 3.5×0.4 mm^2^, respectively.


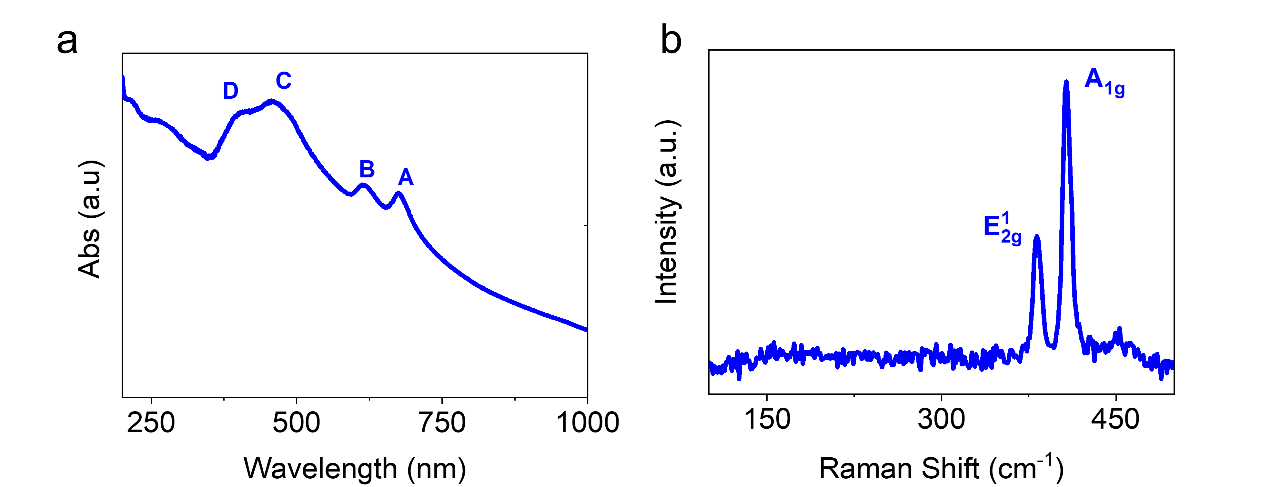


**Figure S2.** (a) UV-Visible spectra of LPE MoS_2_ dispersion. (b) Raman Spectra of LPE MoS_2_ NCs.

**Figure S2a** shows the absorption spectra of the MoS_2_ dispersion, the peaks at 413, 457, 613 and 675 nm are the characteristic absorption peaks of exfoliated MoS_2_ NCs obtained by liquid phase exfoliation. Figure S2b shows a typical Raman spectrum of exfoliated MoS_2_, which exhibits two characteristic peaks, A_1g_ and E_2g_, corresponding to the out-of-plane and in-plane vibration modes of 2H-MoS_2_ layers, respectively.


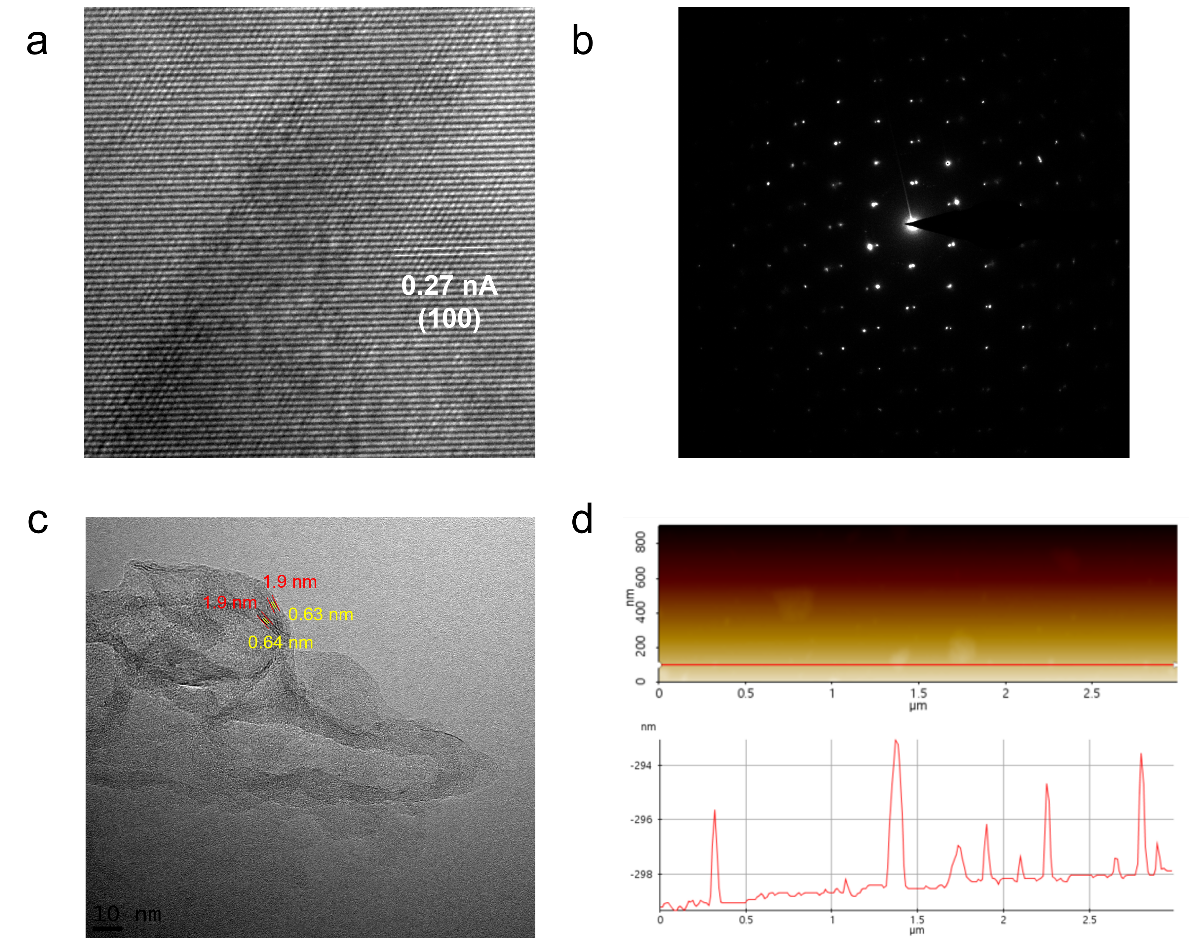
**Figure S3.** (a) HRTEM image of exfoliated MoS_2_ NCs. (b) SEAD image of twist exfoliated MoS_2_ NCs. (c) HRTEM image of MoS_2_ NCs. (d) AFM analysis of MoS_2_ NCs.

MoS_2_ mixed with QDs can be obtained after a 3000 rpm centrifugation process, as shown in Figure S1. **Figure S3a** shows the HRTEM image of twisted MoS_2_ NCs with the corresponding MoS_2_ lattice spacing, and Figure S3b shows the corresponding SEAD image. The hexagonal structure indicates the formation of high-quality MoS_2_ NCs. Figure S3c shows a typical view of the edges of MoS_2_ NCs with clear lattice fringes. The thickness of MoS_2_ is 1.9 nm and a uniform interlayer spacing of ~0.63 nm is clearly observed, which indicates the formation of 3-4 MoS_2_ layers. AFM analysis of MoS_2_ NCs shows the thickness of MoS_2_ NCs is about 2-5 nm.

**
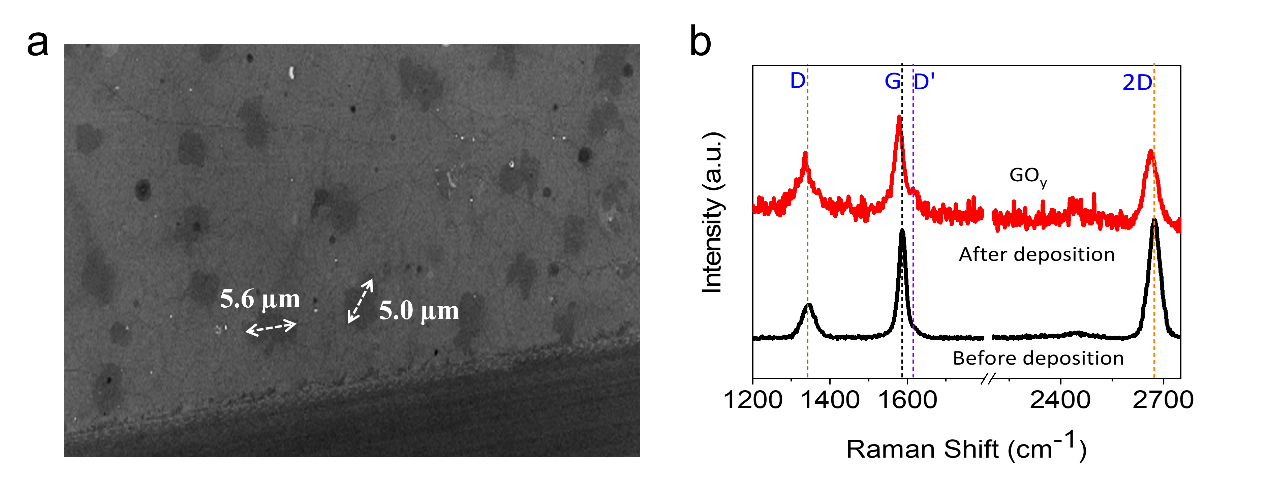
**

**Figure S4.** (a) SEM image of the pristine CVD graphene electrode. (b) Raman spectra of CVD graphene before (black curve) and after (red curve) oxidation;

CVD graphene was grown on Cu foil (99.999%), preliminary annealed at 1060 °C in a hydrogen flow of 300 sccm and an Ar flow of 2000 sccm at a pressure of <10^-4^ Torr for 1-2h in a growth chamber. Graphene was then grown by CVD at 1020 °C from a mixture of CH_4_ (40 sccm) and H_2_ (10 sccm). Finally, the sample was cooled to room temperature under a temperature regime.^1^ **Figure S4a** shows the SEM image of the defective structure, a specific structure distributed over the surface with a mean density of about $3\times{10}^{6}$ cm^-2^ was found. Note that the Raman scattering results for the pristine graphene are calculated on average over 10 points, as shown in Figure S4b (black curve).


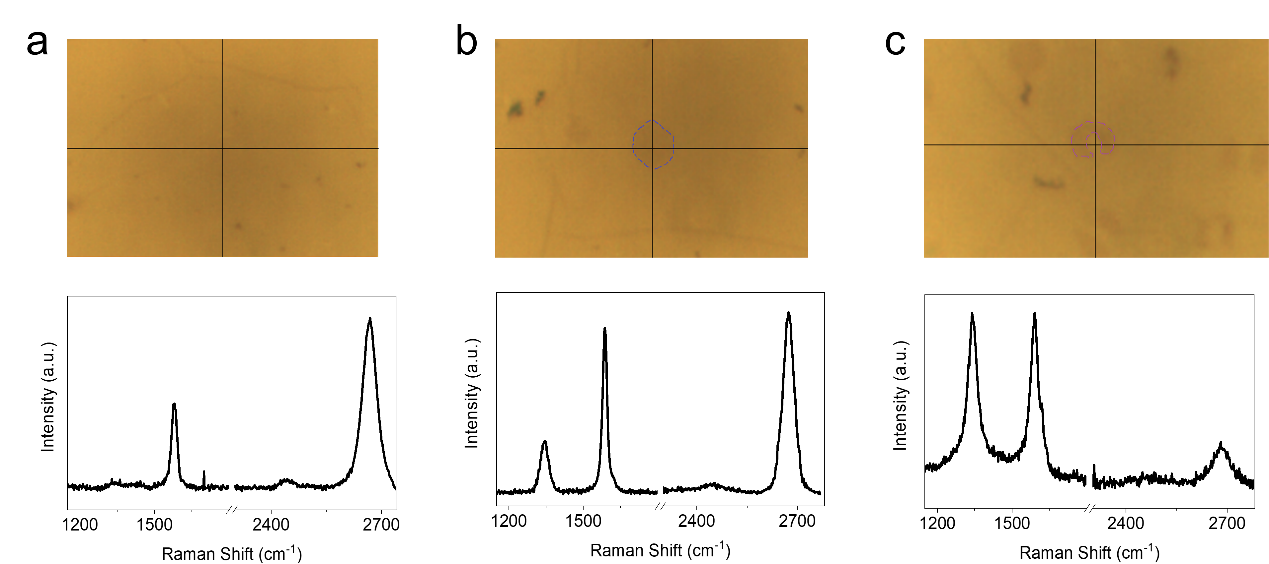
**Figure S5.** Optical images and Raman spectra of a graphene film (a), a multilayer island (b) and an island with a defective region in the center (c).

**Figure S5** shows the corresponding Raman characteristics for a graphene film, a multilayer island and an island with a defective region, respectively. It was found that the main difference between various structures is the rise of I_D_/I_G_, the decrease of I_2D_/I_G_, which indicates the degradation of graphene quality and the formation of a very defective multilayer graphene structure for oxidation and reduction.


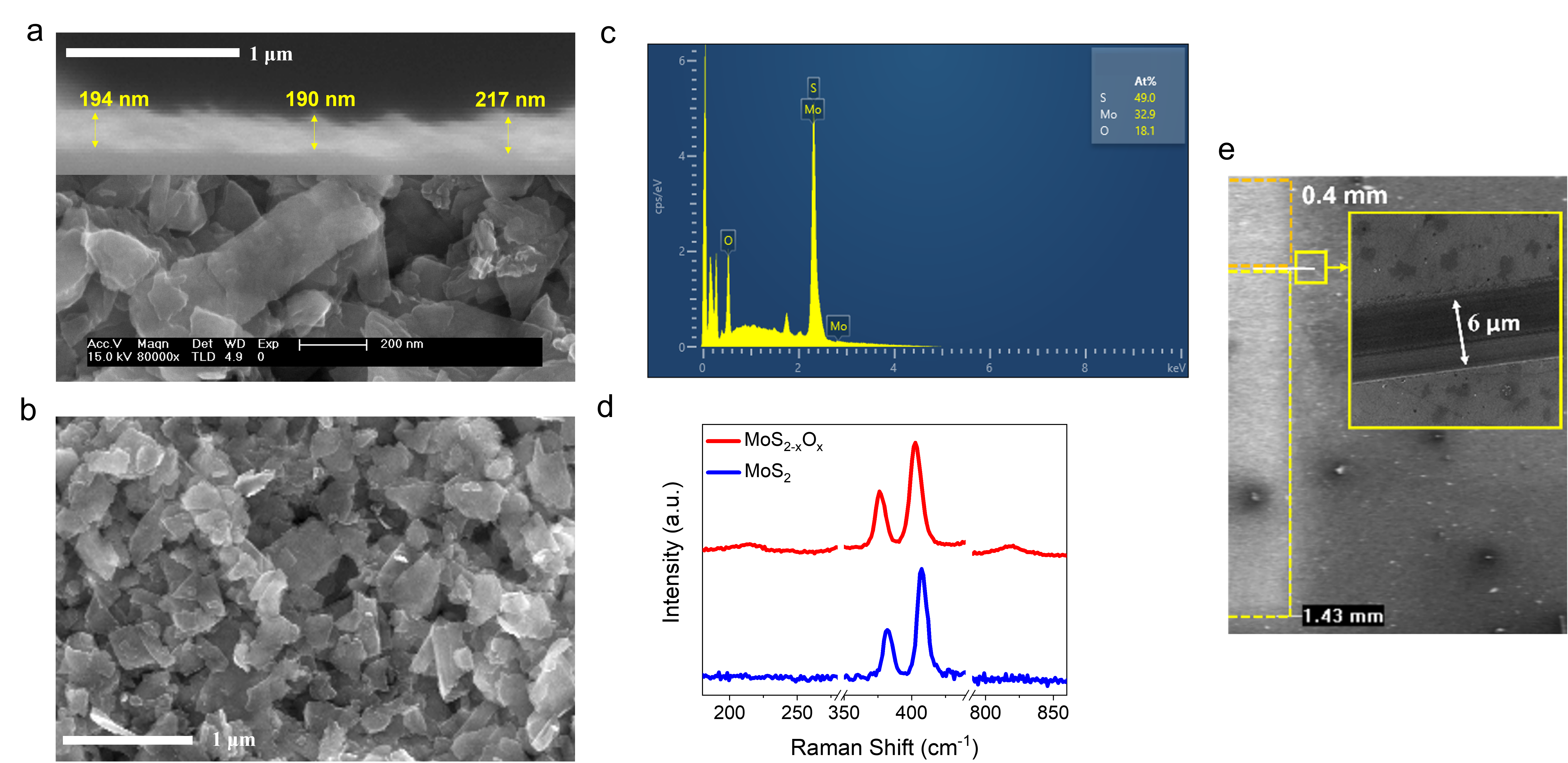
**Figure S6.** (a) SEM image of deposited MoS_(2-x)_O_x_ NCs. The inset shows the SEM image of a cross-section of a MoS_(2-x)_O_x_ thin film obtained on G/SiO_2_. (b) SEM image of a MoS_(2-x)_O_x_ thin film. (c) Analysis of the deposited thin film by energy dispersive X-ray spectroscopy (EDS). (d) Raman spectra of MoS_2_ before (blue curve) and after oxidation (red). (e) SEM image of the edge of a G/M/G device, the channel distance is about 6 um, and the contact area ratio is 1.43:0.4.

After deposition, the MoS_2-x_O_x_ film completely covers the specified area indicated by the mask, with a thickness of more than hundreds of nanometers. As the channels of the devices are micrometers, we have added additional SEM images with scale bars shown in **Figures S6a** and S6b to demonstrate complete coverage and uniformity of MoS_2-x_O_x_ flakes and deposited film thickness. The thickness varies from 190nm to 217 nm.

The deposition of the MoS_2_ film in the surrounding ambient atmosphere leads to the oxidation of the structure. The thickness of the MoS_(2-x)_O_x_ film is 200 nm, it is in contact with two lateral GO_y_, and was formed through a mask with a given asymmetric geometry (x≈0.27). A pulse of -4V for 8 seconds is applied under illumination to initiate the device. As shown in Figure S6d, the presence of small peaks at 220 cm^-2^, 820 cm^-1^ and the blue shift of the $E_{2g}^{1}$and A_1g_ modes indicate the formation of p-type MoS_2-x_O_x_.^2-4^

**Section B: Current-Voltage Measurements**

**
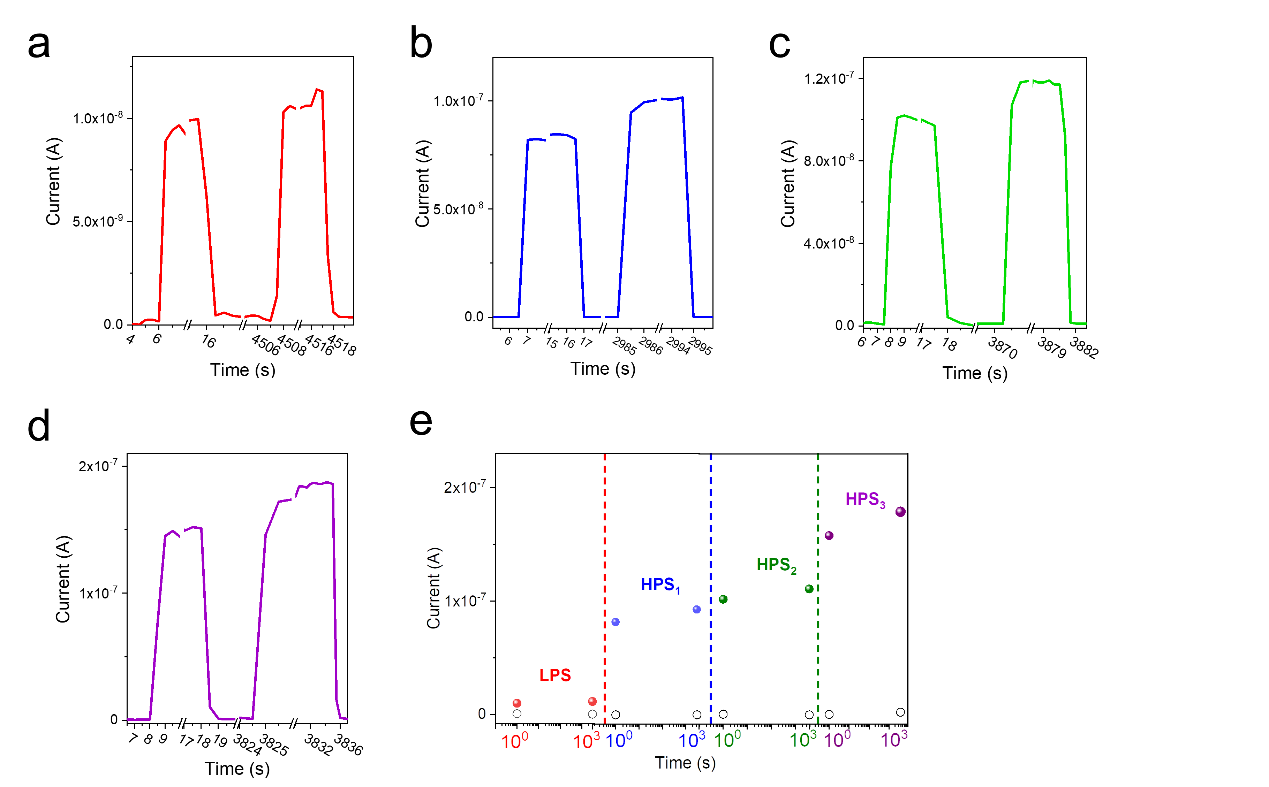
Figure S7.** (a-d) Photocurrent response of the device to light pulses of 10s duration for LPS, HPS_1_, HPS_2,_ and HPS_3_, respectively. (e) Retention times of multilevel photoresponse states at 0 bias, the symbols represent current without illumination- open spheres; photocurrent at LPS, HPS_1_, HPS_2_, HPS_3_-red, blue, green and purple spheres, respectively.

**Figure S7** shows the photoresponse of the photomemristor, The photomemristor is illuminated with a 10 s pulse of light from a solar simulator (power density 56 mW/cm^2^). During the hold, the light was turned off. The retention time for LPS and HPS states is more than 3×10^3^ seconds at room temperature.


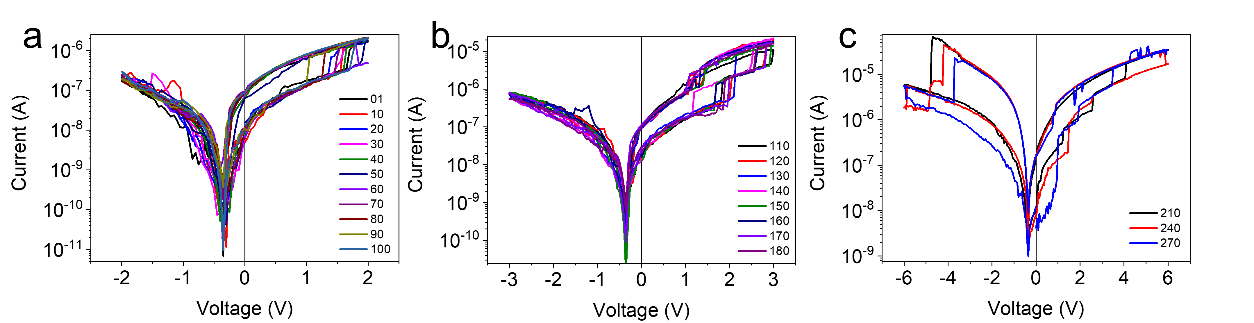
**Figure S8.** I-V characteristics of the G/M/G device in the light for hundreds of sweeps of various bias voltages, which represent hundreds of experimental switching loops for various photoresponse states, indicate a high degree of repeatability of photoresponse state switching.


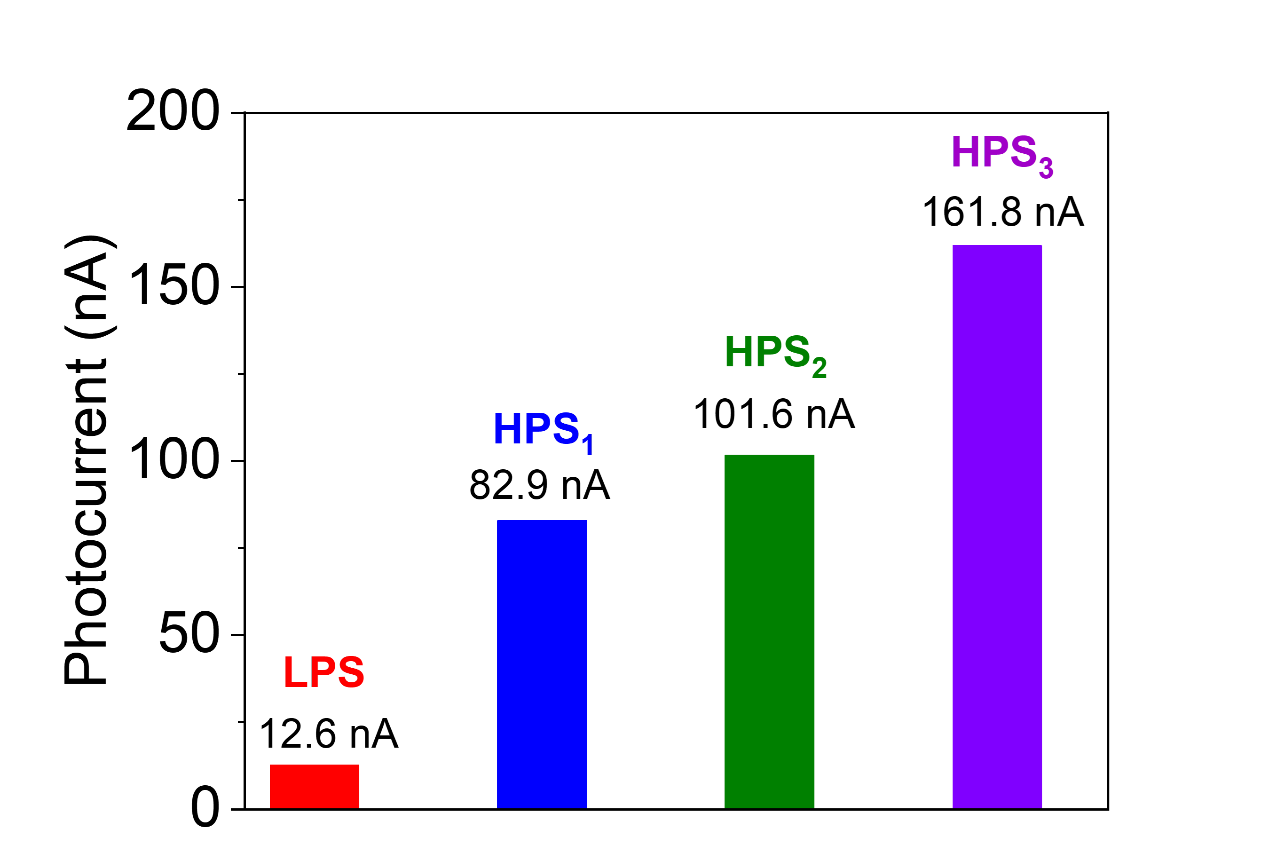


**Figure** **S9**. Photocurrent readout at 0V for various photoresponse states, photocurrent was calculated as the average of hundreds of switching loops at various photoresponse states.


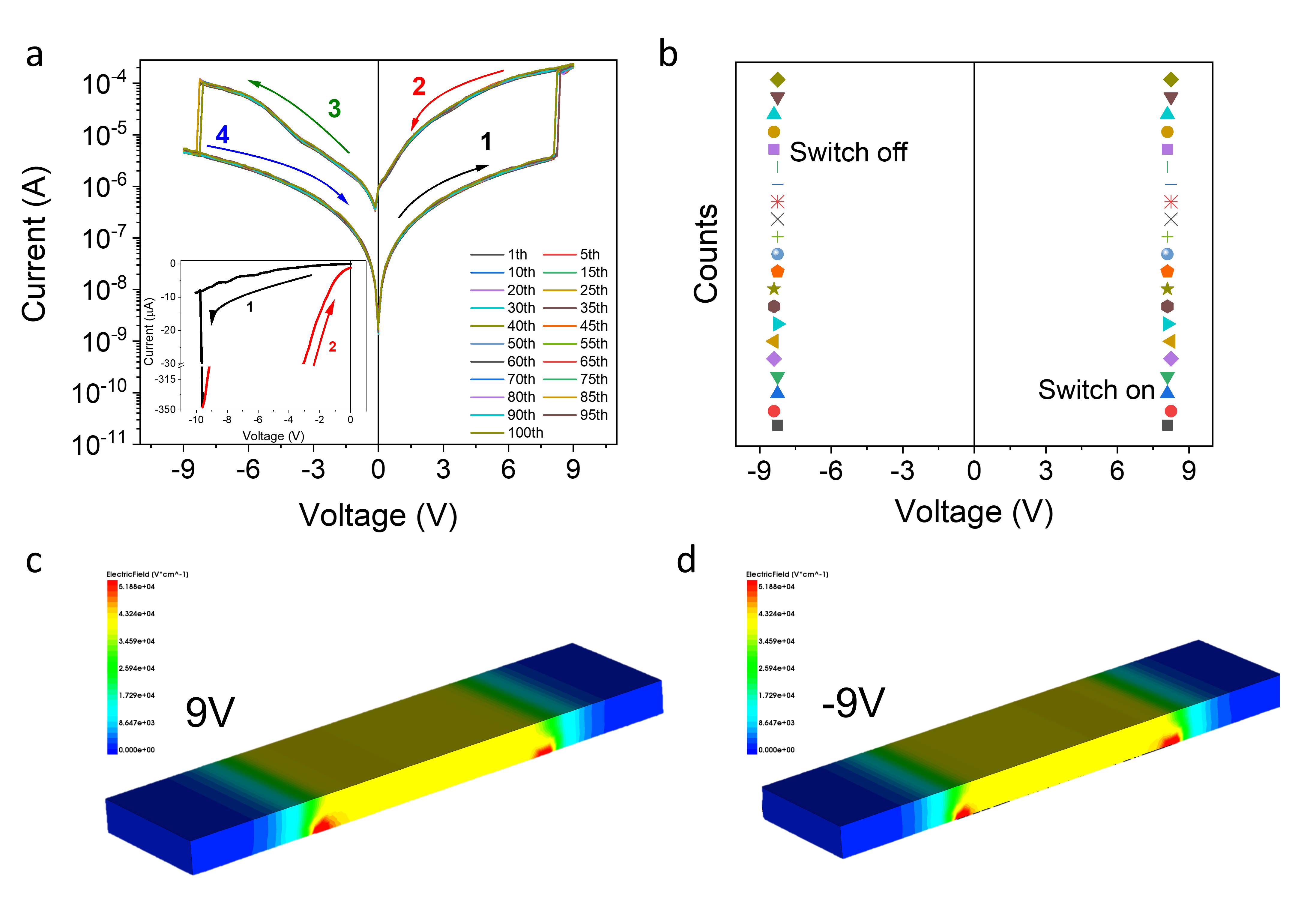
**Figure S10.** (a) Current-voltage characteristics of the switching photoresponse when the voltage sweeps from 0 V to 9 V, from 9 V to -9 V, and back to 0 V under illumination. The inset shows photoresponsive switching for negative short-circuit photocurrent when the voltage sweeps from 0 V to -10.05 V, and back to 0 V under illumination. (b) Change in On/Off voltage during cycling for a G/M/G device. TCAD-simulated electric field of G/M/G devices under a bias voltage of 9 V (c) and -9 V (d) in light.

The switching of the photoresponse is strongly dependent on the voltage distribution across the diode. For a device with a large distance between electrodes working in the low voltage range, we can simplify this structure into two back-to-back Schottky diodes connected in series. When the light is off, the current through the two Schottky diodes should be the same. The voltage drops mainly across the reverse-biased diode. As a result, we cannot obtain a symmetric conductance variation in the dark when the same small voltage pulses are applied. However, under illumination, it's quite different. The reverse photocurrent is in the same order as the forward current in a certain range, because the reverse photocurrent is enhanced due to the effective collection of photo-generated carriers in the depletion region. Thus, the voltage distribution is also similar. This allows us to obtain a reversible photoresponse switching under illumination in a small voltage range because of the symmetrical drift and diffusion of oxygen ions. However, as the states changed, the history of the voltage drop across the two diodes changed again, which led to less stable results.

To overcome this problem, we can apply a large enough voltage to the device with two electrodes to fully deplete the semiconductor between the electrodes. In this case, the electric field is in the same order in the whole channel (TCAD simulation: **Figures S10c** and S10d).^5^ At the same time, we increase the sweep speed by 4 times to avoid low voltage switching, thus the result is optimized.


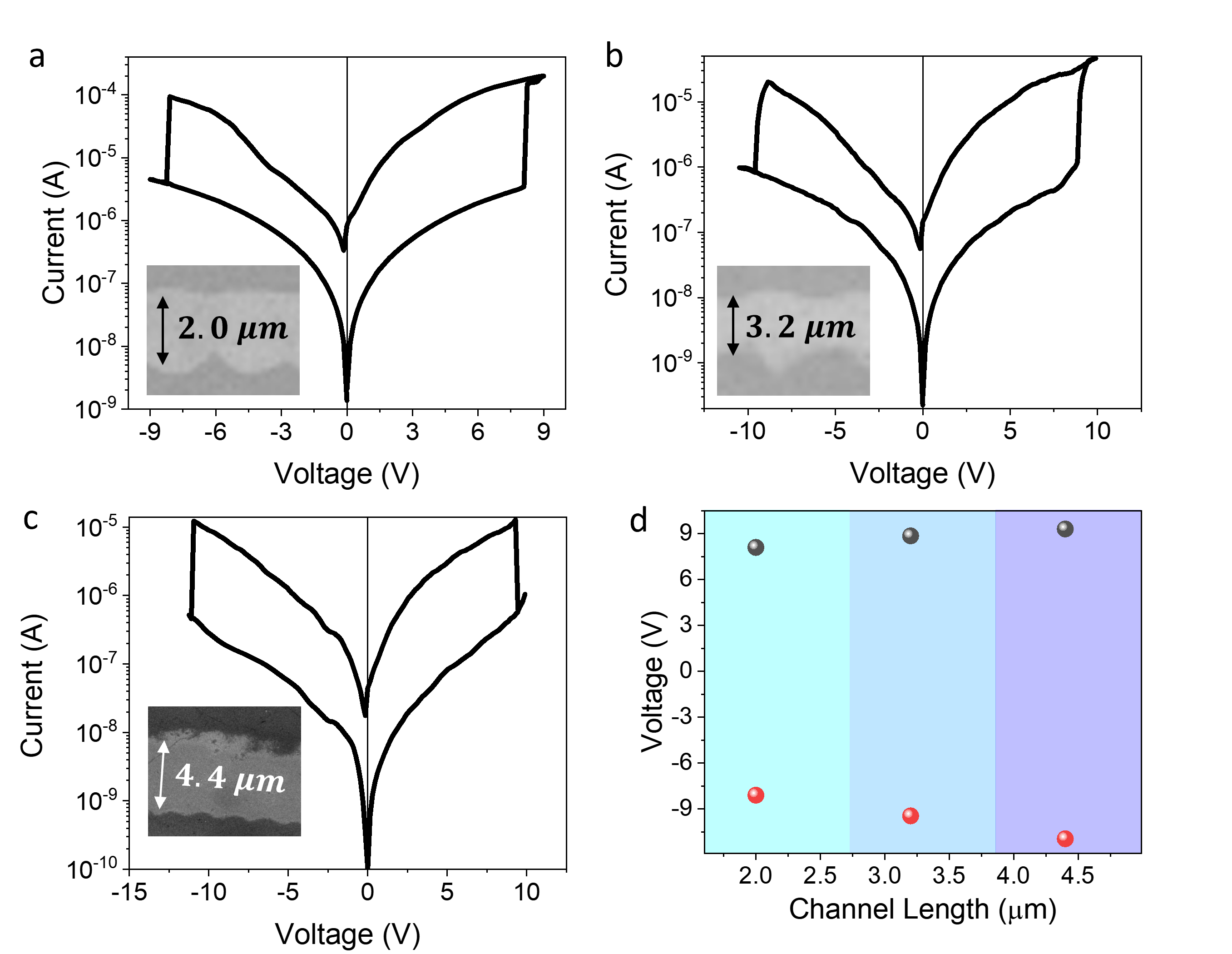


**Figure S11**. (a) I-V characteristics of switching photoresponse when the voltage sweeps from 0 V to 9 V, and from 9 V to -9 V, and back to 0 V under illumination. The inset shows the SEM image of the channel before depositing the MoS_2-x_O_x_ film. (b)-(c). I-V characteristics of G/M/G photomemristors of 3.2 μm and 4.4 μm. The thickness of MoS_2-x_O_x_ in (a)-(d) is 120 nm. (d) Switch On/Off voltage of the devices with different channel lengths.


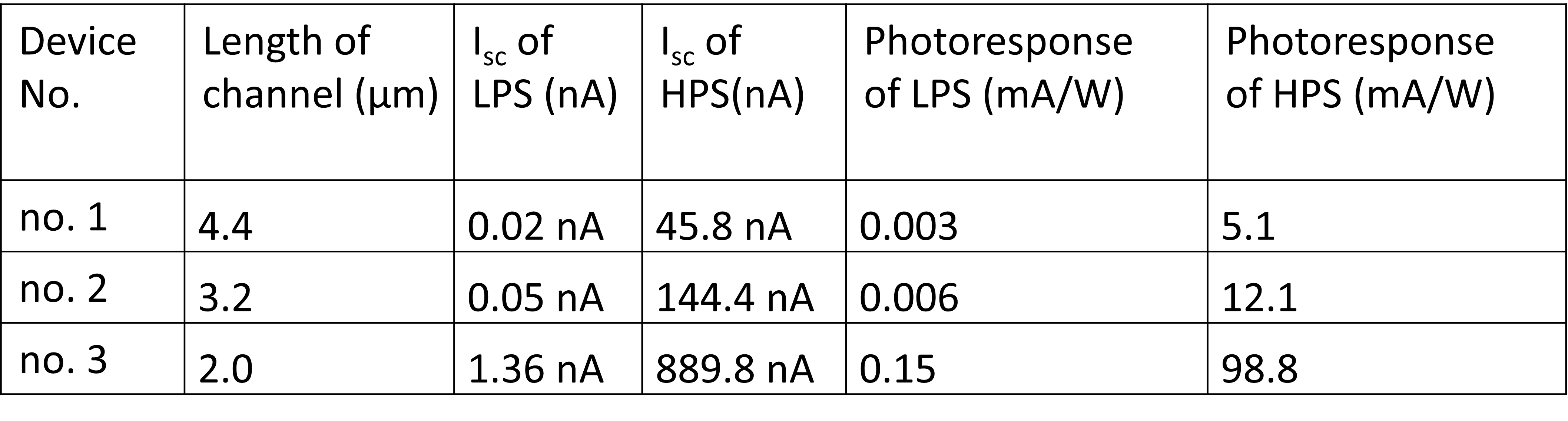


**Table S1**. Typical parameters of G/M/G devices with different sizes.

Table S1 summarized the parameters and performance of different devices, the photoresponsivity (R) of the G/M/G device was calculated based on the following equations:^6^

𝑅 = 𝐼𝑝/(𝑃 × 𝑆) (S1)

where 𝐼𝑝 is the photocurrent, 𝑆 is the active area under illumination, and 𝑃 is the incident light intensity.

The highest tunable short-circuit photocurrent reported in our paper of 889.8 nA is much higher compared with other 2D materials.^7,8^ This value is even higher than that of Si-based tunable short-circuit photocurrent devices.^9^ To obtain a gate tunable short-circuit photocurrent, the semiconductor channel must be thin enough. Thus, its hard to fabricate a thick film to absorb light. In our cases, the mechanism of the two-terminal device is based on ion migration, which does not limit the thickness. We can increase the thickness of the film to absorb more photons.

On the other hand, an atomically thin 2D material can obtain high photo gain due to the photogating effect, but it doesn't work without a bias voltage. Considering the limited light absorption and operating voltage of 0, the photoresponse of gate tunable three/four-terminal devices is also very limited. To the best of our knowledge, the tunable photoresponse of 98.8 mA/W in our manuscript is also the highest value never reported.

**Section C: Raman characterization**


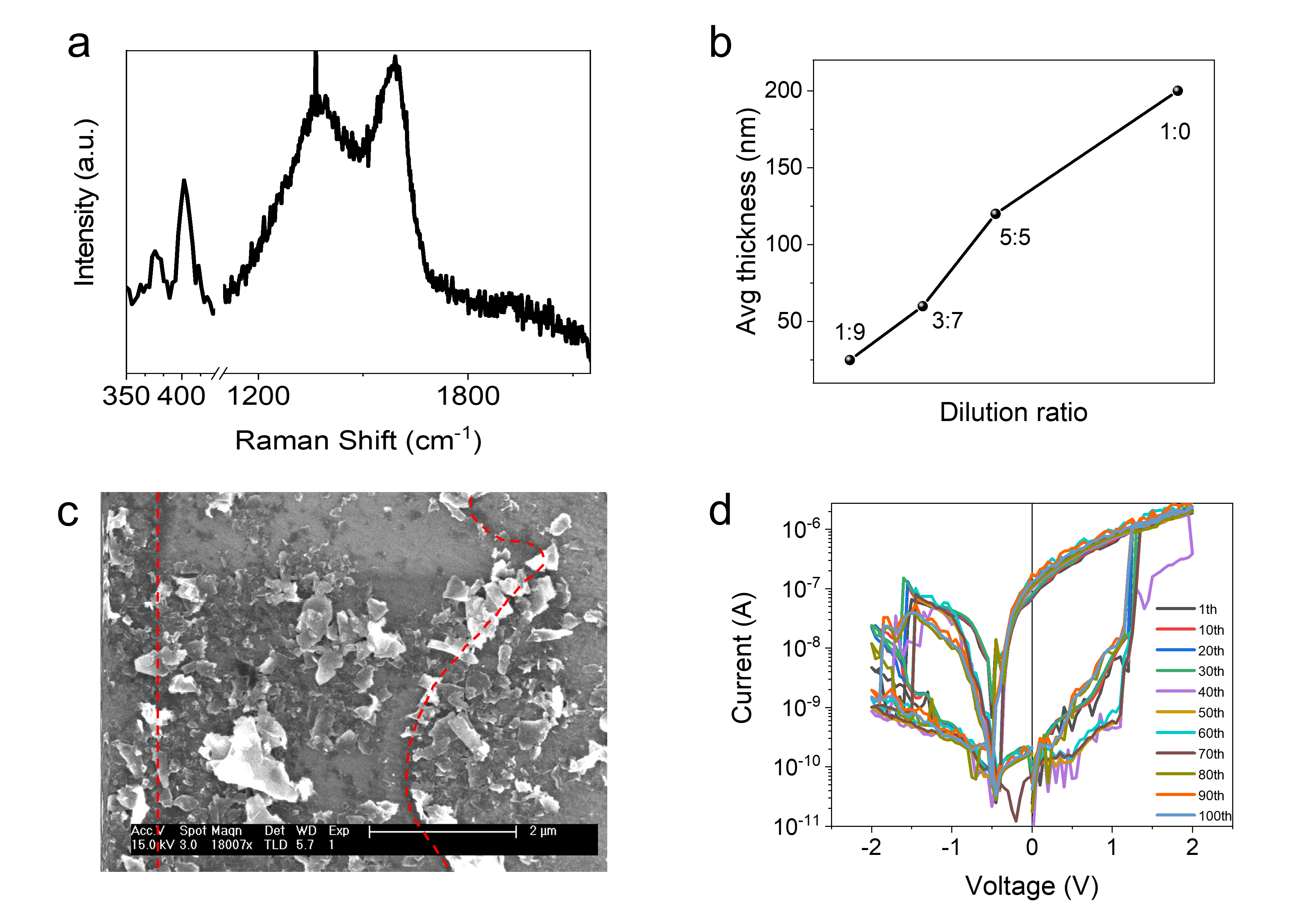


**Figure S12.** (a) Raman spectra of G/M/G with thick MoS_2-x_O_x_. (b) The relationship between the average thickness of MoS_2-x_O_x_ film and the dilution ratio (MoS_2_ dispersion : Solution). (c) SEM image of a specific G/M/G structure for Raman characterization, the channel length is 2 µm. (d) I-V characteristics of specific G/M/G photomemristors with a thinner MoS_2-x_O_x_ film when the voltage sweeps from 0 V to 2 V, from 2 V to -2 V, and back to 0 V under illumination.

Due to the inevitable carbon contamination during the evaporation process, it’s difficult to estimate the feature of graphene from the Raman spectrum when a thick enough MoS_2-x_O_x_ film is deposited. Therefore, the Raman characterization was carried out at a specific point in the thin G/M/G structure for various states.

To obtain thinner MoS_2-x_O_x_, we diluted the dispersion several times, and the relationship between the average thickness of MoS_2-x_O_x_ film and the dilution ratio is shown in **Figure S12b**. We deposited a MoS_2-x_O_x_ film with a thickness of about 25 nm to obtain Raman characteristics.


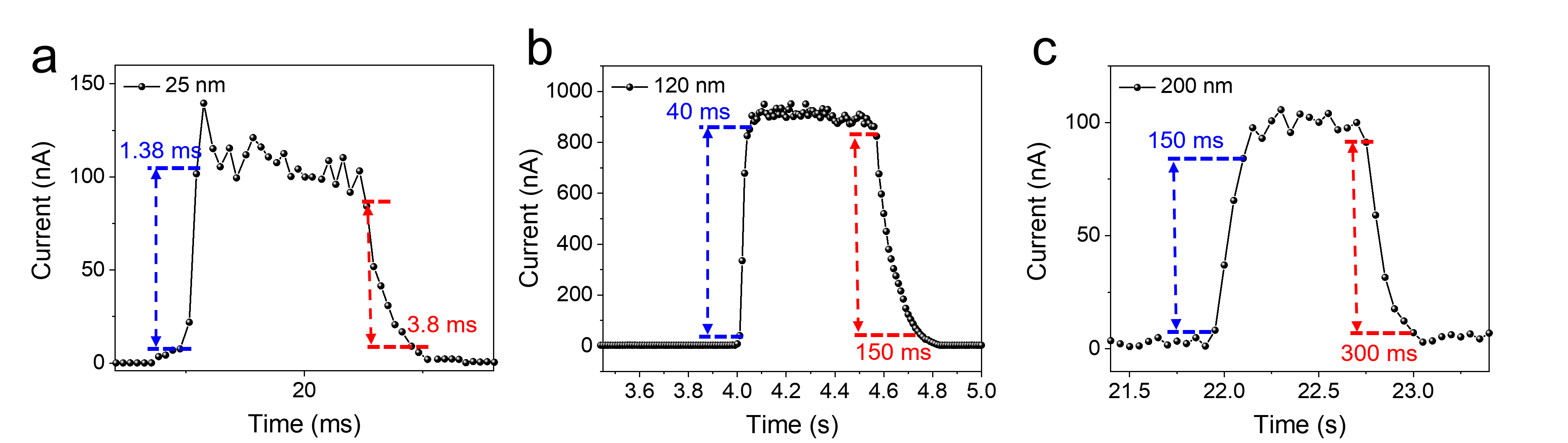
**Figure S13.** The photoresponse of the G/M/G devices was measured at a bias voltage of 0 V under illumination with a light intensity of 56 mW cm^-2^.

The response time decreases when the thickness of the film decreases. This behavior may be attributed to the slow carrier mobility in the vertical direction as the extremely large mobility anisotropy in 2D materials.^10^ The photocarriers formed in the upper layers arrive at the lower graphene electrodes with a significant delay for a thick MoS_2-x_O_x_ sample. In much thinner devices, the response time is much faster.


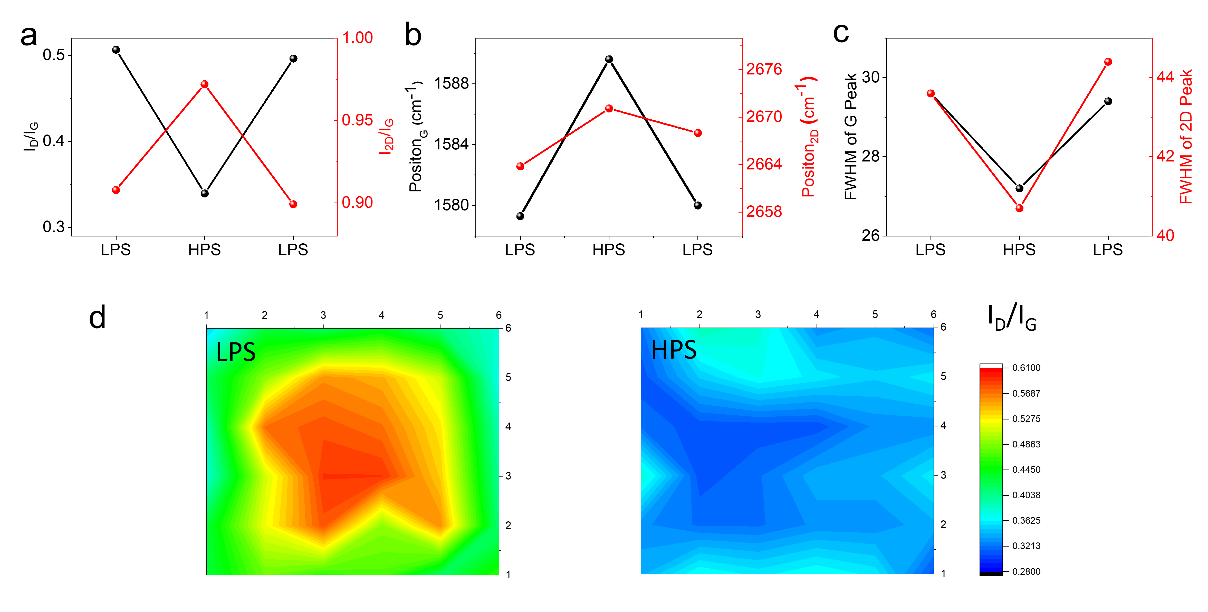
**Figure S14.** Correlation changes in the cathode in the states of the photoresponse of the device for (a) Raman intensity ratios for G- and 2D-modes; (b) Shifts of the G and 2D modes; (c) Full width at half maximum (FWHM) of the G peak and 2D peak. (d) Raman mapping of the graphene cathode in LPS and HPS for the same point.

Pristine graphene shows a D/G and 2D/G intensity ratio of around 0.3 and 1.2, respectively. With the increment of the degree of lattice disorder after oxidation, the ratio of the defect-induced D band and the graphite-originated G band (I_D_/I_G_) increases from 0.3 to 0.5, while the 2D/G intensity ratio decreases from 1.2 to 0.9. When the device switches from LPS to HPS, the ratio of I_D_/I_G_ decreases from 0.5 to 0.3, the ratio of I_2D_/I_G_ increases from 0.9 to 1.0; the peak position of the G band and the 2D band shows a slight red shift and a decrease in the FWHM of the G mode and the 2D mode can be observed as shown in **Figure S14b-c**, corresponding to a reduction process of GO_y_ according to the previous reports^11,12^. After the Reset process, the ratio of I_D_/I_G_ increases up to 0.5, and the ratio of I_2D_/I_G_ decreases to 0.9; a slightly blue shift and broadening of the G band and the 2D band can be observed, which indicates the oxidation process^11-13^.

To demonstrate the variation of I_D_/I_G_ clearly, we provide Raman mapping counts from 36 points of the specific point on the graphene electrode. Figure S14d clearly shows the decrease in the ratio of I_D_/I_G_ after the reduction.


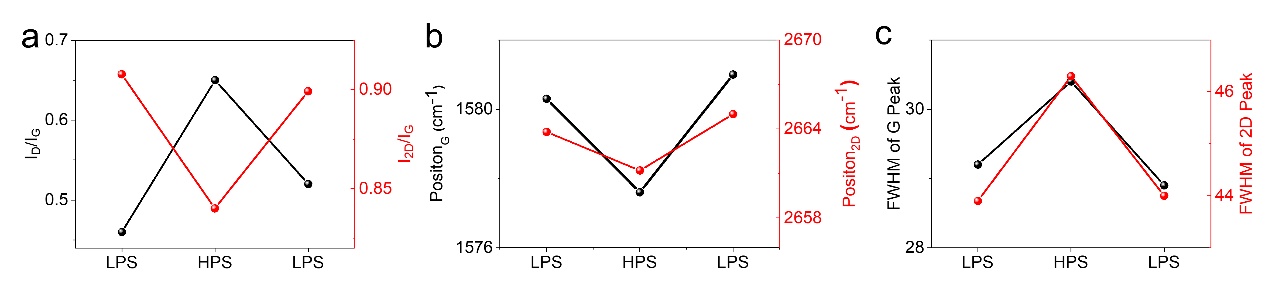
**Figure S15.** Correlation changes in the anode of the states of the photoresponse of the device for: (a) Raman intensity ratios for G- and 2D-modes; (b) Shifts of the G and 2D modes; (c) Full width at half maximum (FWHM) of the G peak and 2D peak.

| Material parameters | | Optical parameters | |
| --- | --- | --- | --- |
| Doping concentration | 1e17 cm^-3^ | Wavelength | 520 nm |
| Electron affinity | 4.3 eV | Intensity | 100 W/cm^2^ |
| Channel length | 2 μm | Model | Ray tracing |
| Barrier height | 0.38 eV | Number of rays | 30000 |

**Table S2**. Material and optic parameters for TCAD simulations

The Synopsys Sentaurus Device was used for the TCAD simulation. Sentaurus Device predicts the behavior of devices by discretizing device architecture and information to solve it in the partial differential equation form. The ray tracing optical model in Sentaurus Device is used to calculate the photovoltaic phenomenon of the device. The light intensity was fixed at 100 W/cm^2^ and the wavelength at 520 nm. The barrier height of our model structure is 0.38 eV for electrical analysis (CVD graphene work function: 4.7-4.8 eV;^14^ and MoS_2-x_O_x_ ionization potential: 5.08-5.09 eV.^2^). Modeling with a ray tracing optical model solves sets of coupled differential equations, namely the Poisson’s equation, the continuity equations for electrons and holes, and the current continuity equations for electrons and holes and current equations for electrons and holes in three dimensions.

**
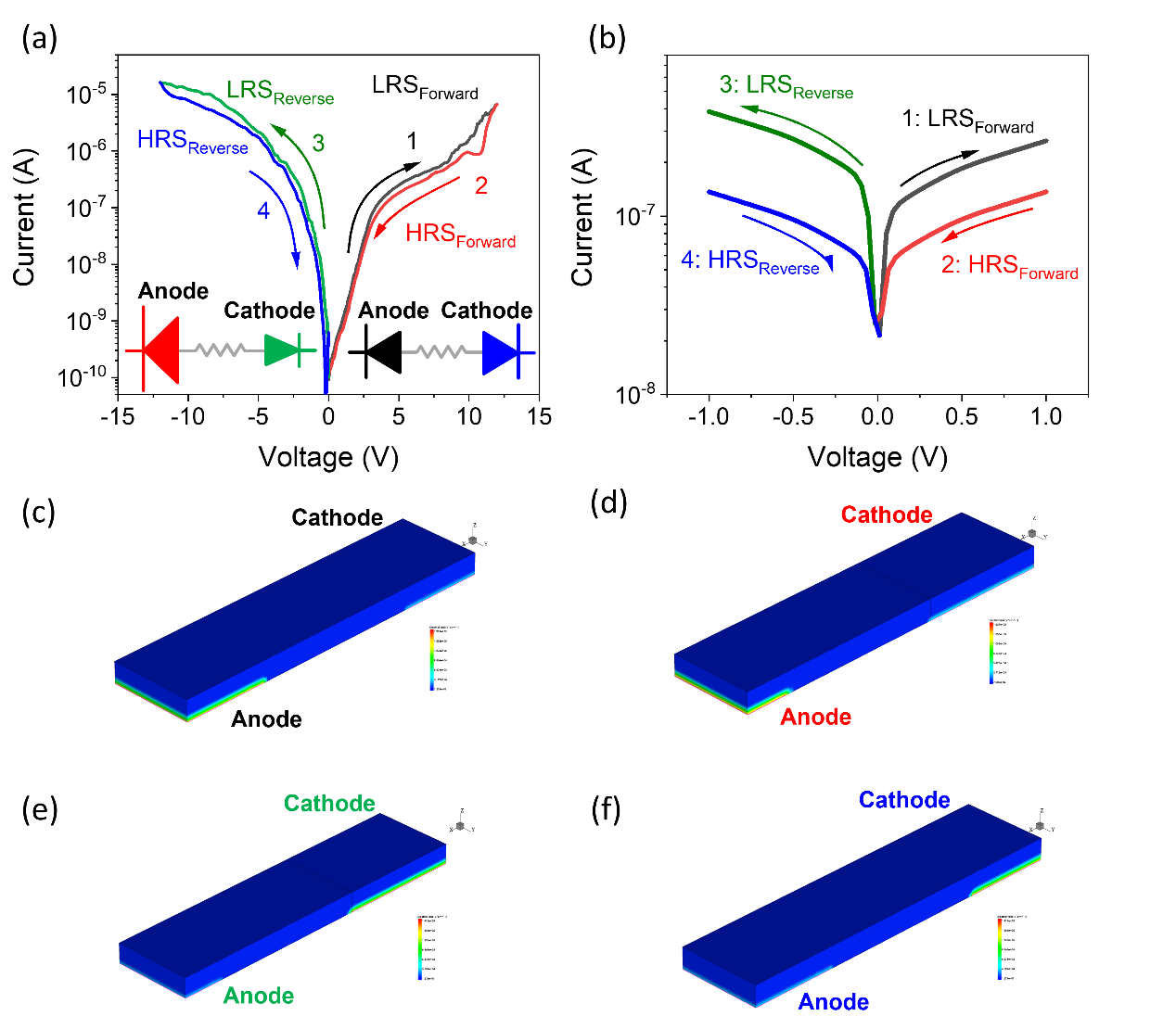
Figure S16**. (a) I-V curves of the G/M/G device when the voltage sweeps from 0 V to 12 V, and from 12 V to -12 V, and back to 0 V in the dark. (b) TCAD simulation of four states (LRS_Forward_, HRS_Forward_, LRS_Reverse_ and HRS_Reverse_) for a G/M/G device. TCAD-simulated electric field for four states at a bias of 1V for LRS_Forward_ (c), 1V for HRS_Forward_ (d), -1V for LRS_Reverse_ (e), -1V for HRS_Reverse_ (f).

**Figure S16** shows the resistive switching behavior of the G/M/G device. When we read a G/M/G device at low voltage, the device act as two back-to-back Schottky diodes connected in series, as shown in the inset of Figure S16a. When the contact size increases (decreases), the conductance of the corresponding diode increases (decreases), and we simplify this change by the diode size. We describe the Set and Read process below:

**Read LRS_Forward_:**

We read the device at a low voltage of 1V, in this case, the voltage is mainly dropped across the reverse diode (black diode near anode as shown on the right side of Figure 16a). In the state of LRS_Forward_, the corresponding TCAD-simulated I-V curve and electric field distribution at 1V are shown in Figure S16b (black curve) and Figure S16c.

**Set:**

To obtain reliable resistive switching, 12 V was applied to completely deplete the channel. When a positive voltage is applied, the migration of oxygen ions (vacancies) toward the anode (cathode) oxidizes (reduces) the anode (cathode). The effective contact size of the anode (cathode) decreases (increases) similarly to Figure 2.

**Read HRS_Forward_:**

The device is read at a low voltage of 1V. In this case, the voltage drops mainly across the reverse diode (the red diode near the anode, as shown on the left side of Figure S16a). The current is lower than the current in LRS_Forward_ due to the smaller contact area. In the HRS_Forward_, the corresponding TCAD-simulated I-V curve and electric field distribution at 1V are shown in Figure S16b (red curve) and Figure S16d.

**Read LRS_Reverse_:**

The device is read at a low voltage of -1V. In this case, the voltage drops mainly across the green reverse diode near the cathode as shown on the left side of Figure S16a. The current is larger because of the bigger contact area. In the LRS_Reverse_, the corresponding TCAD-simulated I-V curve and electric field distribution at 1V are shown in Figure S16b (green curve) and Figure S16e.

**Reset:**

When a negative voltage is applied, the migration of oxygen ions (vacancies) toward the cathode (anode) oxidizes (reduces) the cathode (anode). The effective size of the anode (cathode) increases (decreases).

**Read HRS_Reverse_:**

The device is read at a low voltage of -1V. In this case, the voltage drops mainly across the blue reverse diode near the cathode as shown on the right side of Figure S16a. The current is lower than the current in LRS_Reverse_ due to the smaller effective contact area. In the state of HRS_Reverse_, the corresponding TCAD-simulated I-V curve and electric field distribution at 1V are shown in Figure S16b (blue curve) and Figure S16f.


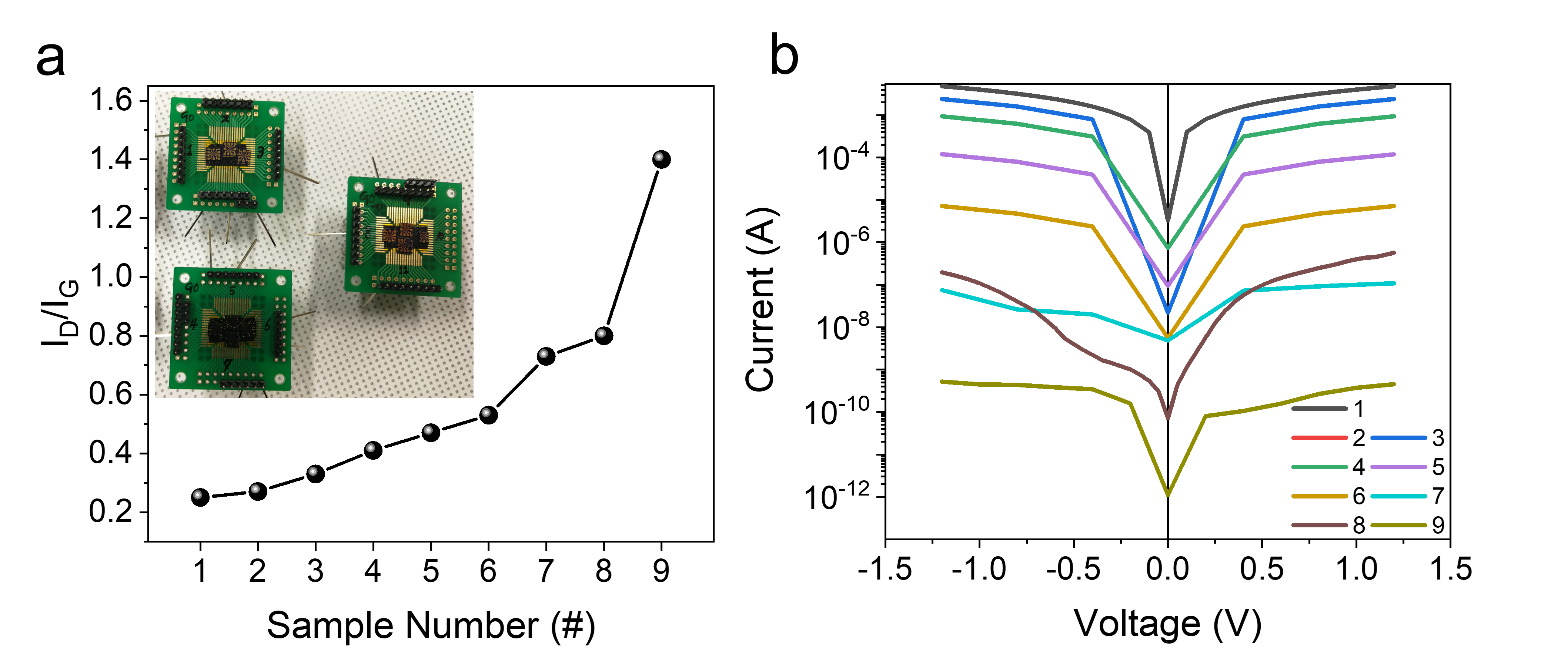


**Figure S17**. (a) I_D_/I_G_ ratio for CVD graphene with different degrees of oxidation. The inset shows an optical image of these 9 samples. (b) I-V characteristics of CVD graphene containing structural defects with different degrees of oxidation.

**Figure S17** shows the I-V and Raman characteristics of CVD graphene containing structural defects with different degrees of oxidation. The I_D_/I_G_ ratio increases when exposure time in an O_2_ riched atmosphere increases (Figure S17a). To estimate the conductivity of graphene after oxidation, we prepared 9 devices with Au/Graphene/Au (A/G/A) structure as shown in the inset of Figure S17a. The conductivities of these 9 devices are shown in Figure 17b. The electrical conductivity decreases when the oxidation degree increases.


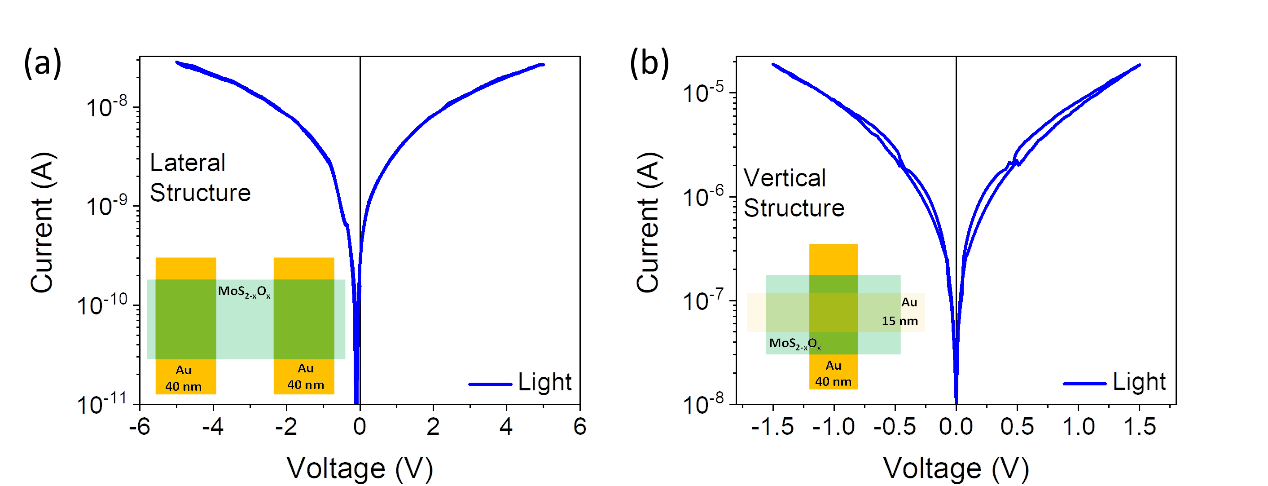
**Figure S18**. I-V curves measured under illumination for the lateral structure (a, voltage sweep from 0 V to 5 V, from 5 V to -5 V, and back to 0 V) and the vertical structure (b, voltage sweep from 0 V to 1.5 V, from 1.5 V to -1.5 V, and back to 0 V). The insets show schematic representations of the devices.

We present data from a study of a similar device with Au electrodes. Such devices have not shown reliable non-volatile photoresponsive switching under illumination. These results show that the oxidation and reduction of graphene electrodes play an important role in the non-volatile photosensitivity switching for our device. In addition, graphene as a two-dimensional crystal has unique optoelectronic and mechanical properties that are attractive for neuromorphic vision devices, where the structure studied in this article can be used.

**Section D: Implementation of stateful logical operations with photomemristors**


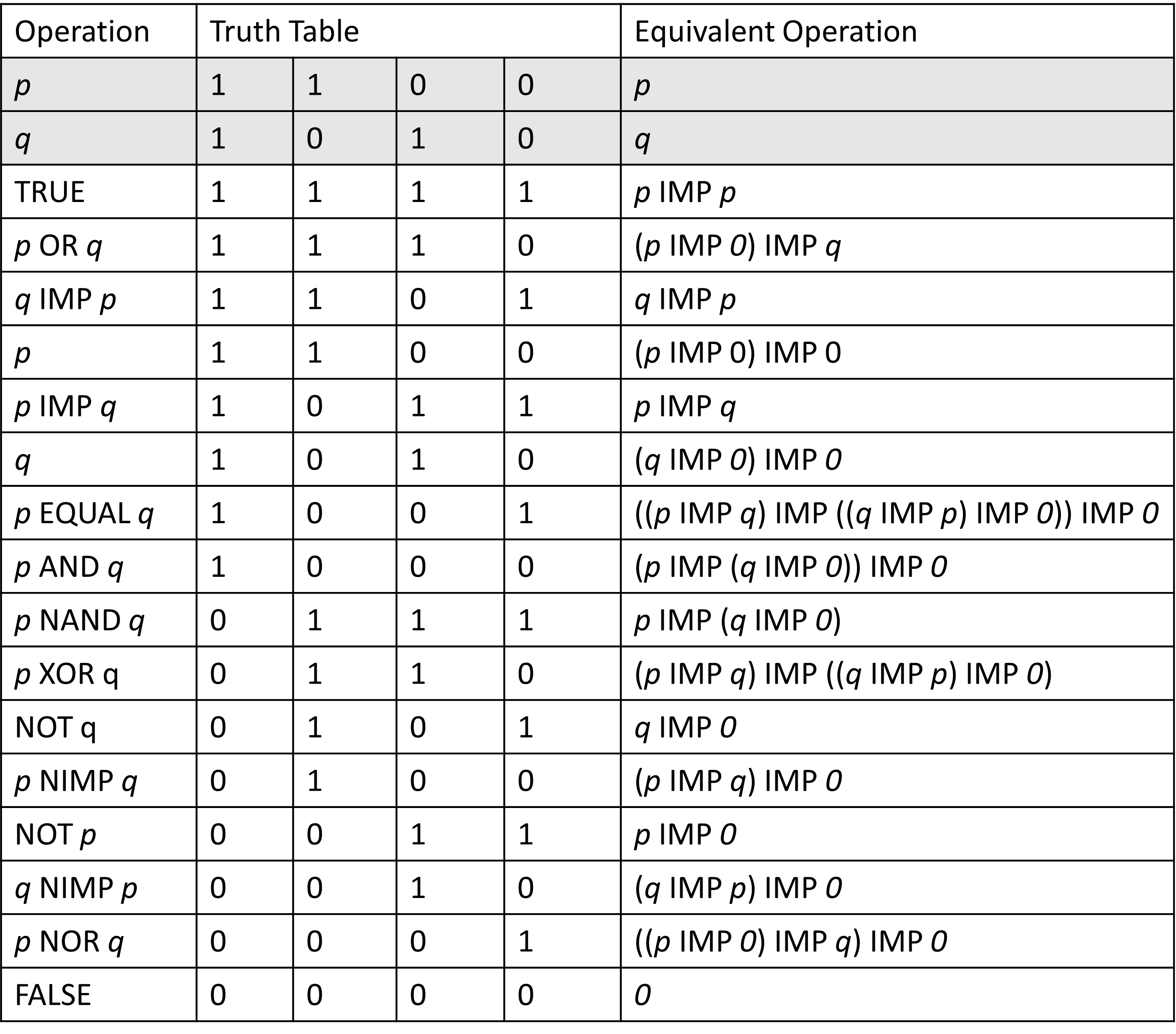


**Table S3**. Computational Universality of IMP and FALSE Operations: the 16 distinct binary Boolean operations on two logic values.^15^


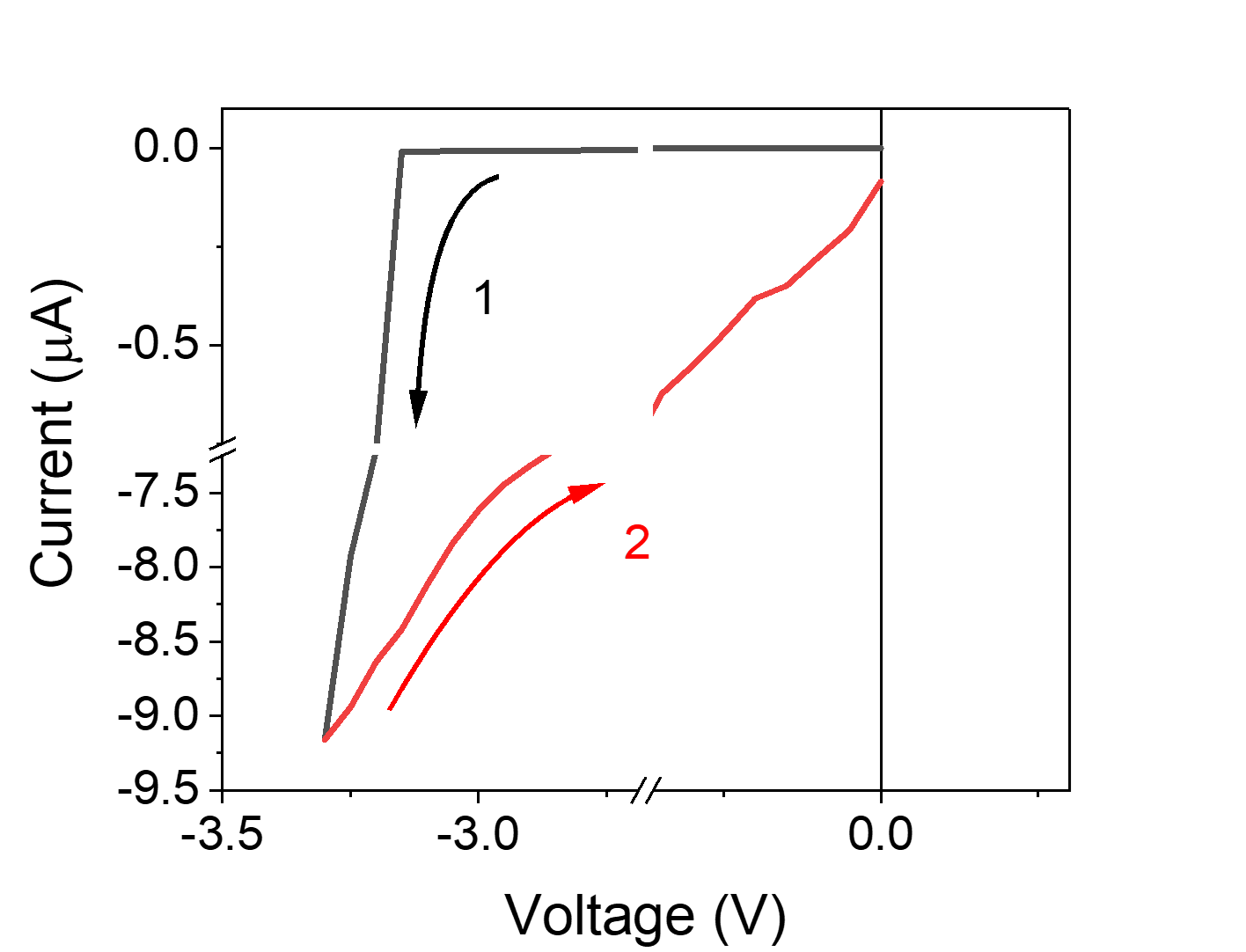
**Figure S19**. I-V curves of the photomemristor when the voltage sweeps from 0 to -3.3 V, and back to 0 V when illuminated.

**Section E: Emulation of a retinomorphic vision sensor**

**
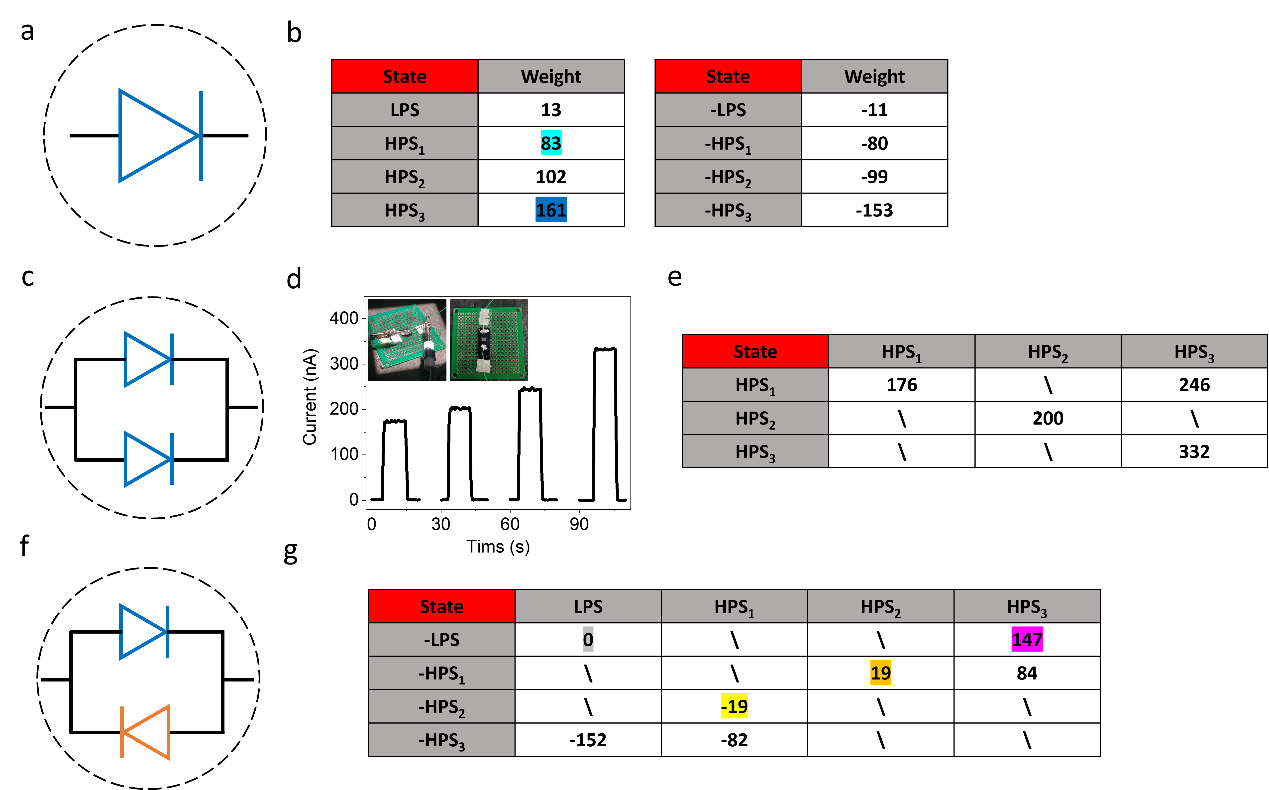
**

**Figure S20.** (a) Schematic representation of a single photomemristor cell for various photoresponse states. (b) Photocurrent (power density 56 mW/cm^-2^) for LPS, HPS_1_, HPS_2_, and HPS_3_ reading at 0V and its opposite polarity, the unit in the Table is nA. (c) Schematic representation of photomemristors connected in parallel with the same direction for various photoresponse states. (d-e) The photocurrent response of devices in (c) to light pulses (power density 56 mW cm^-2^) of 10s duration for HPS_1_ & HPS_1_, HPS_1_ & HPS_3_, HPS_2_ & HPS_2,_ and HPS_3_ & HPS_3_, respectively. The inset of Figure S13d shows the two devices assembled with wire bonding. The unit in the Table is nA. (f) Schematic representation of photomemristors connected in parallel with the opposite direction for various photoresponse states. (g) The short-circuit current of devices in (f) to light pulses (power density 56 mW/cm^-2^) of 10s duration. The unit in the Table is nA.

As the short-circuit photocurrent can hardly increase with higher voltage stimuli, the multi-photoresponse states can be realized by varying the different states and polarities of the photomemristors. By setting the photoresponse state of each photomemristor with different polarities, the output photocurrents of -11, -80, -99, -153 nA can be obtained as shown in **Figures S20a** and 20b. By connecting two devices in parallel, we can get short-circuit currents of 176 nA, 200 nA, 246 nA, 332 nA with the same polarity of the two devices. When the polarities of the two devices are reversed, the current in the circuit is -152 nA, -82 nA, -19 nA, 0 nA, 19 nA, 84 nA or 147 nA as shown in Figure 5b and Figure S20g. Note that we only retain the photoresponse states which generate distinguishable circuit currents.

**
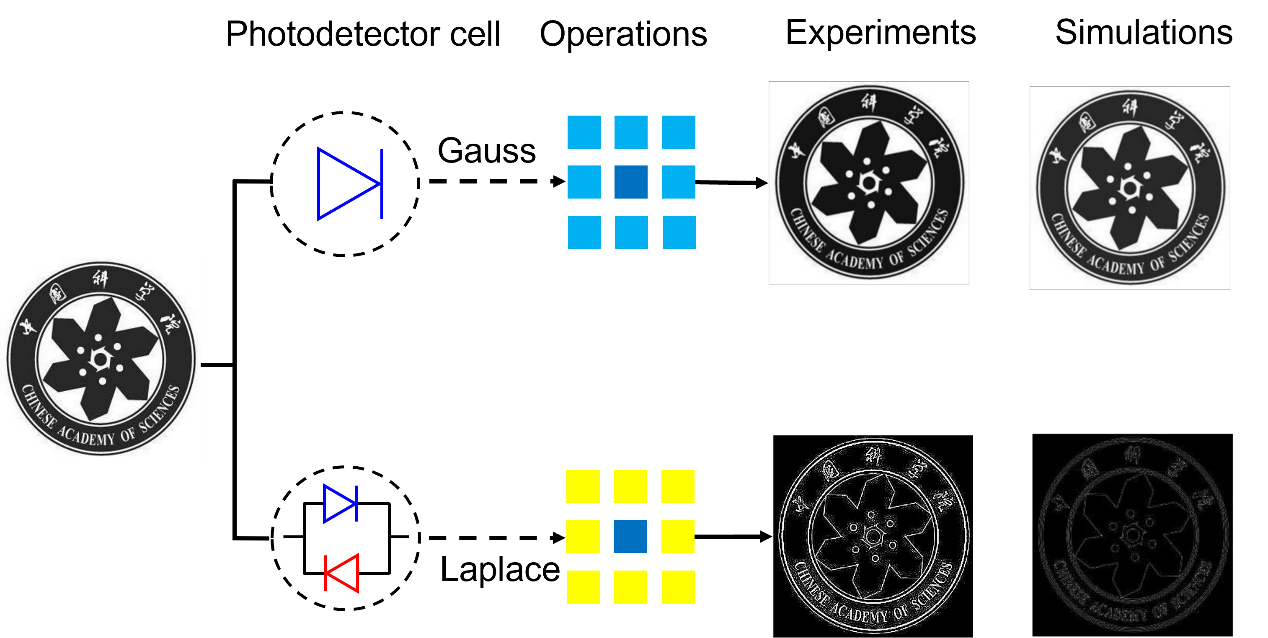
Figure S21.** Demonstration of image pre-processing with the photomemristor array.

The operation of Gaussian blur is emulated by a $3\times3$ photo-memristor array with the individual device written to a specific photoresponse state, in which each cell contains a single photomemristor as shown in **Figure S21**. The Experiment results emulated from the photoresponse states matrix of $\left[ \begin{matrix} 83 & 83 & 83 \\ 83 & 161 & 83 \\ 83 & 83 & 83 \end{matrix} \right]$ with our photomemristor, while the simulated blurred image was calculated with a matrix of $\left[ \begin{matrix} 1 & 1 & 1 \\ 1 & 2 & 1 \\ 1 & 1 & 1 \end{matrix} \right]$. The operation of the Laplacian operator is emulated by a $3\times3$ photomemristor array with two photomemristors written to a specific photoresponse state. The experiment results were emulated from the photoresponse state matrix of $\left[ \begin{matrix} -19 & -19 & -19 \\ -19 & 147 & -19 \\ -19 & -19 & -19 \end{matrix} \right]$ with our photomemristor set, while the simulated blurred image was calculated with a matrix of $\left[ \begin{matrix} -1 & -1 & -1 \\ -1 & 8 & -1 \\ -1 & -1 & -1 \end{matrix} \right]$.


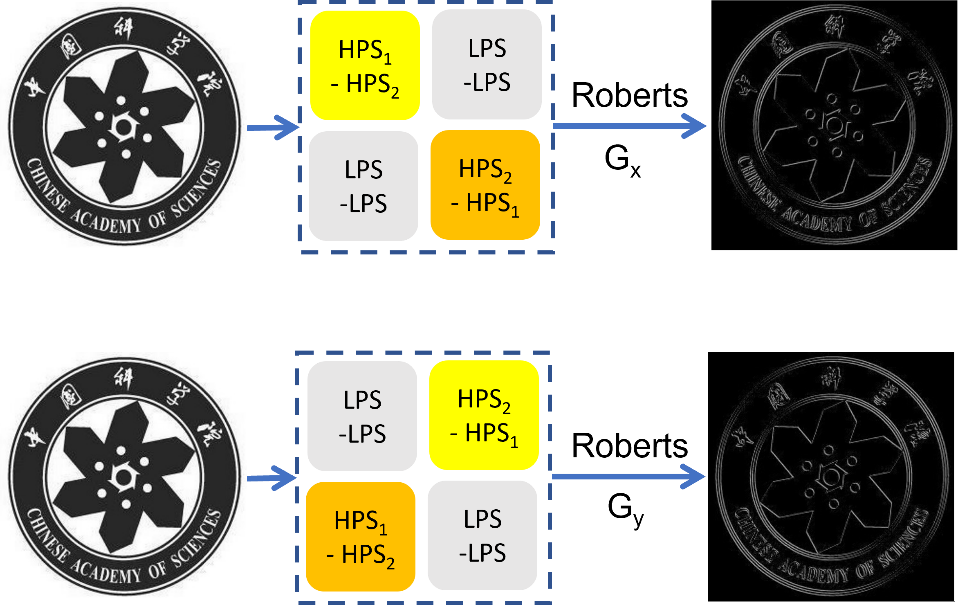


**Figure S22**. Demonstration of image pre-processing of Roberts operator with the photomemristors array.

The operation of image pre-processing of the Roberts operator is emulated by $2\times2$ photomemristor arrays, with a single device being written to a specific photoresponse state in which each cell contains 2 photomemristors, as shown in **Figure S22**.


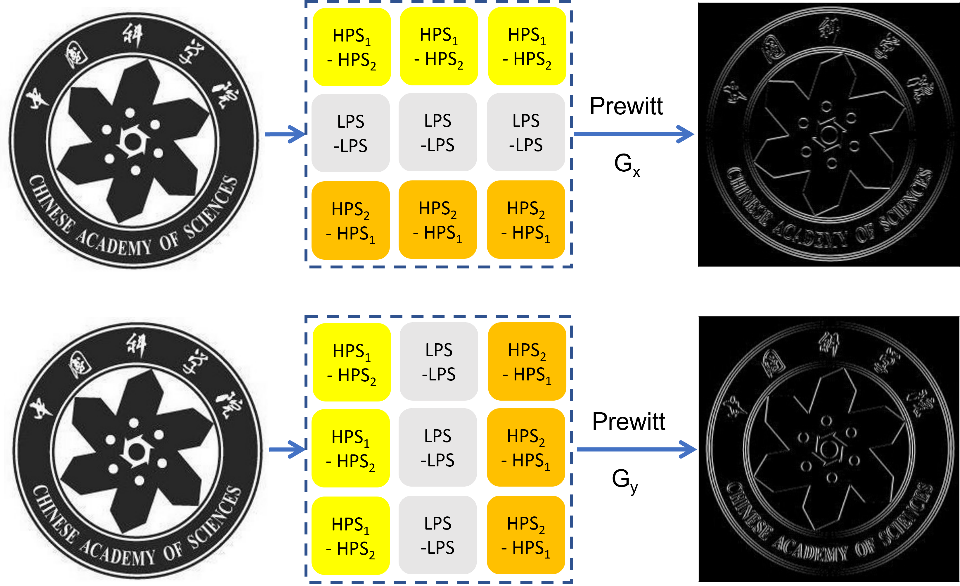


**Figure S23.** Demonstration of image pre-processing of Prewitt operator with an array of photomemristors.

The Prewitt operator image pre-processing operation is emulated by a $3\times3$ photomemristor array with a separate device written to a certain photoresponse state, in which each cell contains 2 photomemristors, as shown in **Figure S23**.


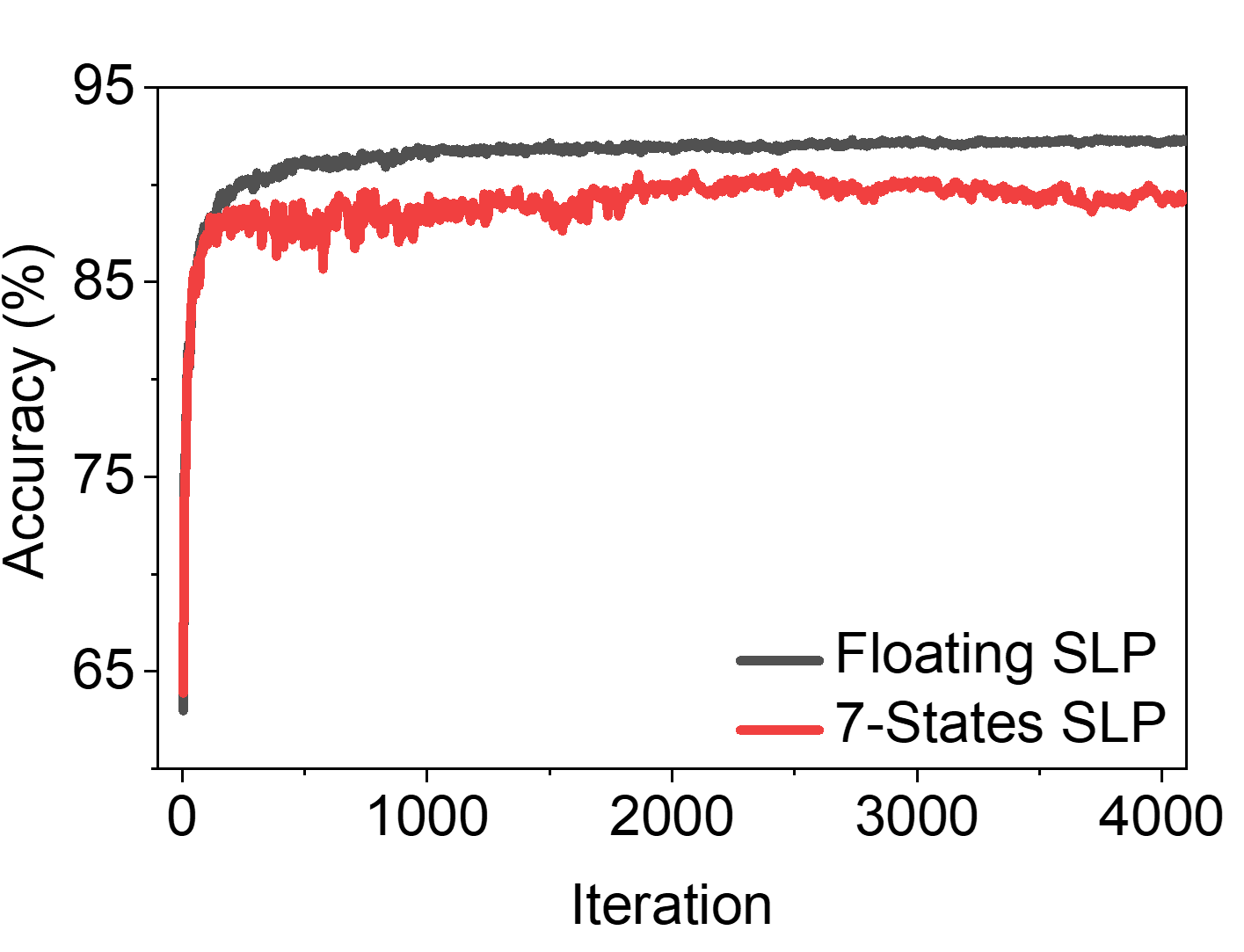


**Figure S24**. Accuracy of the SLP classifier during training with a floating-point weight and a discrepant 7-level photoresponse state.

Such an SLP classifier also allows classifying MNIST data set into 10 classes, when 10 photomemristors can be integrated into one cell. For such a neural network, it is implemented in Pytorch on an NVIDIA TITAN XP GPU. The SLP is trained offline with 60000 images of the training set with a batch size 64 and 4000 iterations, delivering the final output probability that classifies the input image to 10 classes on the testing set (10000 images) with 92.02% accuracy (**Figure S24**). The weights in the FC are discretized to accommodate the 7-levels photoresponse states. After discretization, the accuracy is about 90.03% which is 2% lower than the pristine SLP


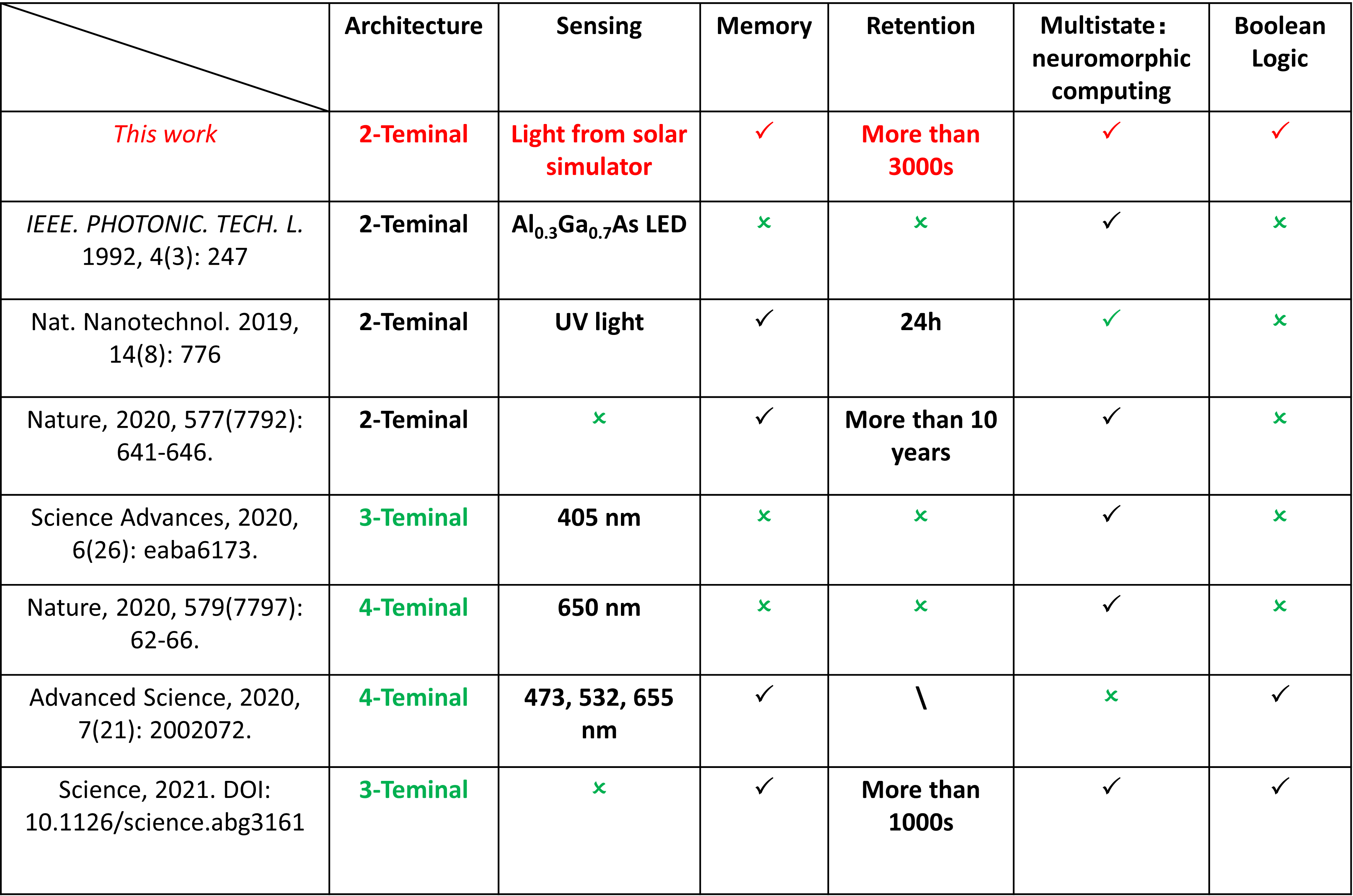
**Table S4**. Typical parameters of neuromorphic computing devices.

We have experimentally demonstrated a memphotoresponsitive switching device with a simple two-terminal architecture in which photoexcited carriers and ion migration are coupled under photoexcitation and bias voltage (**Table S4**). These results are an example of a pinched current-voltage characteristic hysteresis beyond zero under illumination. The theory of the photomemristor and the experimentally obtained characteristics of this new device require further study and establishment. Photomemristors in integrated circuits of artificial neural networks have enormous potential for significantly expanding the functionality of optoelectronic systems, if the theory of photomemristor is well established, and the dynamic nature of such optoelectronic devices is comprehensively studied and effectively used. Imperative applications include ultra-dense in-memory sensing and computing systems, as well as photoinduced learning networks.

| Thickness | Switch on voltage | Switch off voltage |
| --- | --- | --- |
| <100 nm | Uncontrollable | Uncontrollable |
| 120 nm | 7.5V ~ 8.7V | -7.8V ~ -8.85V |

**Table S5.** Switching voltage for G/M/G devices with different thicknesses (channel length: 2 μm)

Because the nanosheets were made by liquid phase exfoliation, the film was deposited using a simple humidifier. When the thickness of the deposited film is less than 100 nm, the device-to-device variation is inevitable and the switching voltage varies from 1.1 V to 2.5 V with a sweeping speed of 0.05 V/s, and 5.1 V to 8.8 V with a sweeping speed of 0.2 V/s. When the thickness of the film is more than 100 nm and working with a high voltage range with a sweep speed of 0.2 V/s, the switching voltages for various samples are more stable.

**References:**

^1^ Kapitanova, O. O. *et al.*, Laterally Selective Oxidation of Large-Scale Graphene with Atomic Oxygen. *The Journal of Physical Chemistry C* **121** 27915 (2017).

^2^ Bhattacharjee, S. *et al.*, Hole injection and rectifying heterojunction photodiodes through vacancy engineering in MoS2. *ADV ELECTRON MATER* **5** 1800863 (2019).

^3^ Neal, A. T., Pachter, R. & Mou, S., P-type conduction in two-dimensional MoS2 via oxygen incorporation. *APPL PHYS LETT* **110** 193103 (2017).

^4^ Wu, S. *et al.*, High-performance p-type MoS2 field-effect transistor by toroidal-magnetic-field controlled oxygen plasma doping. *2D MATER* **6** 25007 (2019).

^5^ Sze, S. M., Coleman Jr, D. J. & Loya, A., Current transport in metal-semiconductor-metal (MSM) structures. *SOLID STATE ELECTRON* **14** 1209 (1971).

^6^ Fu, X. *et al.*, Geometry-asymmetric photodetectors from metal–semiconductor–metal van der Waals heterostructures. *MATER HORIZ* **9** 3095 (2022).

^7^ Pi, L. *et al.*, Broadband convolutional processing using band-alignment-tunable heterostructures. *NAT ELECTRON* **5** 248 (2022).

^8^ Mennel, L. *et al.*, Ultrafast machine vision with 2D material neural network image sensors. *NATURE* **579** 62 (2020).

^9^ Jang, H. *et al.*, In-sensor optoelectronic computing using electrostatically doped silicon. (2022).

^10^ Dragoman, M. *et al.*, A SnS2-based photomemristor driven by sun. *J APPL PHYS* **123** 24506 (2018).

^11^ Mulyana, Y., Uenuma, M., Ishikawa, Y. & Uraoka, Y., Reversible Oxidation of Graphene Through Ultraviolet/Ozone Treatment and Its Nonthermal Reduction through Ultraviolet Irradiation. *The Journal of Physical Chemistry C* **118** 27372 (2014).

^12^ Kwon, S. *et al.*, Reversible oxidation states of single layer graphene tuned by electrostatic potential. *SURF SCI* **612** 37 (2013).

^13^ Kapitanova, O. O., Panin, G. N., Cho, H. D., Baranov, A. N. & Kang, T. W., Formation of self-assembled nanoscale graphene/graphene oxide photomemristive heterojunctions using photocatalytic oxidation. *NANOTECHNOLOGY* **28** 204005 (2017).

^14^ Yoon, T., Wu, Q., Yun, D., Kim, S. H. & Song, Y. J., Direct tuning of graphene work function via chemical vapor deposition control. *SCI REP-UK* **10** 9870 (2020).

^15^ Borghetti, J. *et al.*, 'Memristive' switches enable 'stateful' logic operations via material implication. *NATURE* **464** 873 (2010).
